# Supplementary material for: Long-read direct RNA sequencing reveals epigenetic regulation of chimeric gene-transposon transcripts in Arabidopsis thaliana
Source: Nat Commun. 2023 Jun 5;14:3248. doi: 10.1038/s41467-023-38954-z (PMC10241880; doi:10.1038/s41467-023-38954-z)
Supplement: Supplementary file 1 — Supplementary Information [file 41467_2023_38954_MOESM1_ESM.pdf]

Supplementary Information for "Long-read direct RNA sequencing reveals epigenetic regulation of chimeric gene-transposon transcripts in *Arabidopsis thaliana*" by Berthelier et *al*.

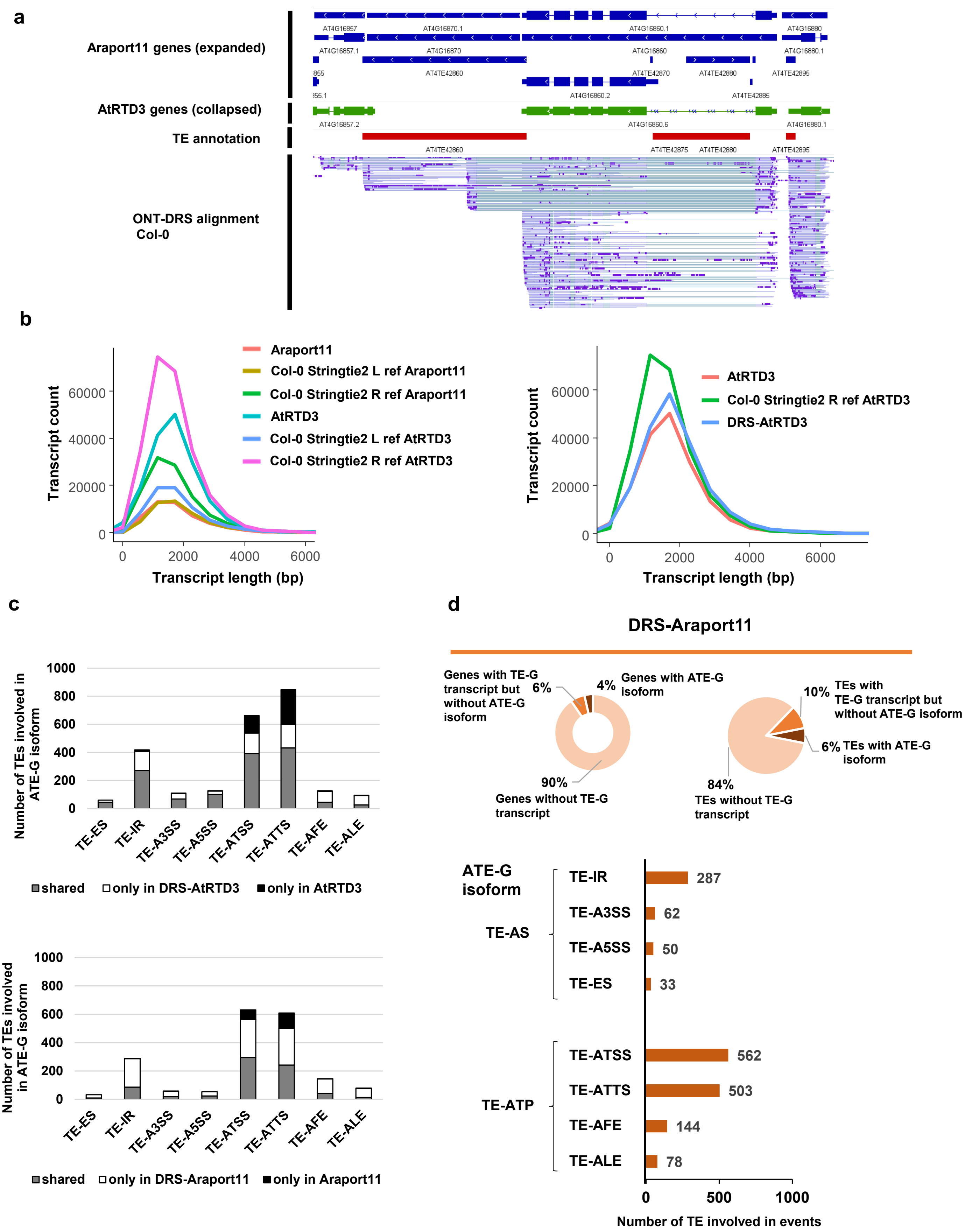

Supplementary Fig. 1. Detection of TE-G transcripts and ATE-G isoforms in the *Arabidopsis* transcriptome. **a)** Representative TE-G transcripts associated with the *RPP4-ATCOPIA4* locus. ONT-DRS reads were aligned to the TAIR10 genome assembly of *A. thaliana*. **b)** DRS transcriptome assembly. Left: the number and length of transcripts obtained from *de novo* assembly of Col-0 ONT-DRS data with “L” or “R” mode in Stringtie2 using Araport11 or AtRTD3 as references. Right: DRS-AtRTD3 transcriptome contains longer and a higher number of transcripts than the original AtRTD3 data. The "Col-0 Stringtie2 R ref AtRTD3" transcriptome contains more transcripts and are, on average, shorter than those from DRS-AtRTD3, reflecting fragmentation of the transcriptome. **c)** Number of TEs involved in ATE-G isoforms according to the RNA processing events, detected by ParasiTE using AtRTD3, Araport11, DRS-AtRTD3, or DRS-Araport11 as a reference. **d)** Top: Number of genes (left) and TEs (right) associated with TE-G transcripts and ATE-G isoforms identified in DRS-Araport11. Bottom: RNA processing events associated with ATE-G isoforms (TE-AS and TE-ATP), and the number of events identified by ParasiTE with DRS-Araport11. Source data are provided as a Source Data file.

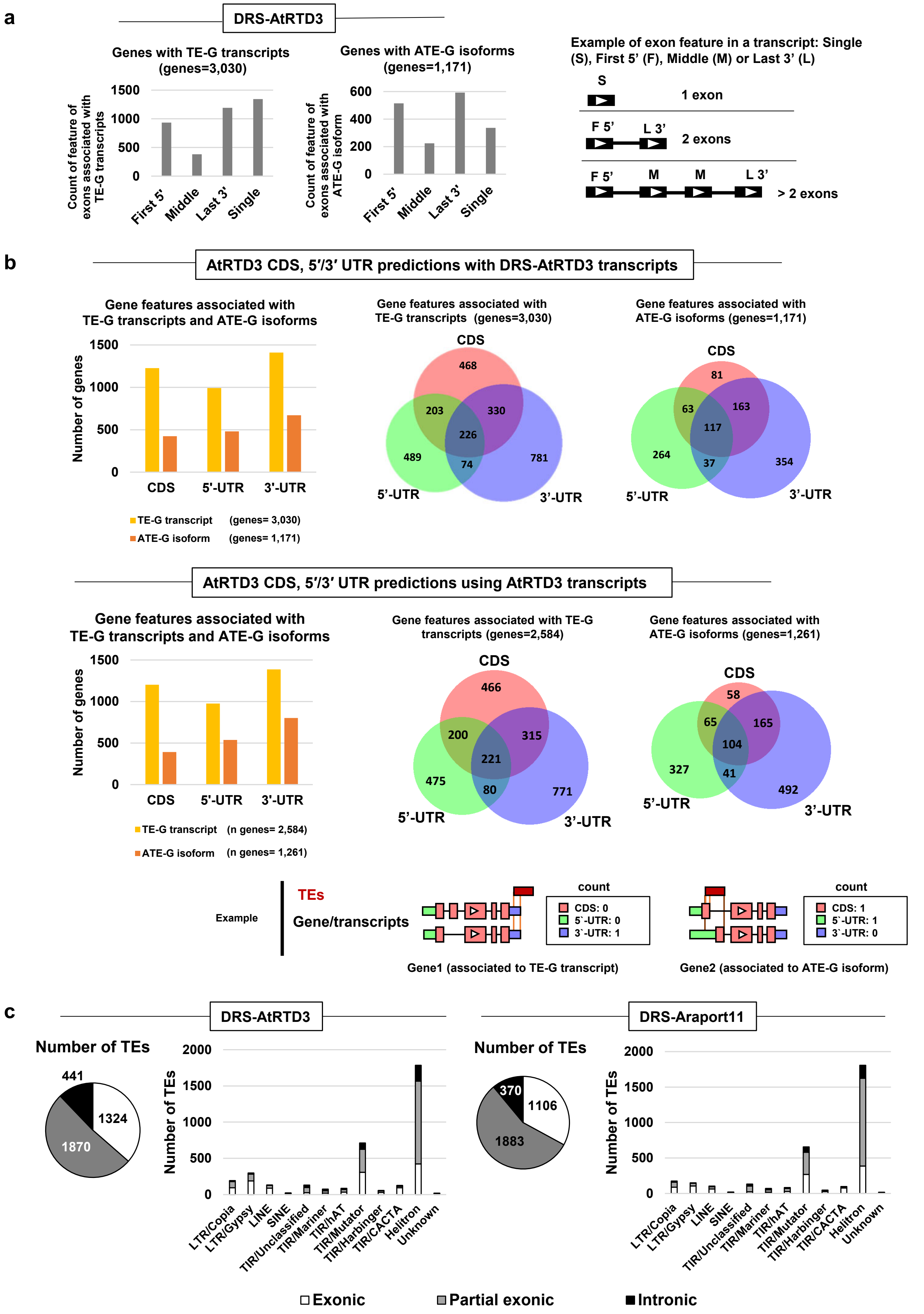

Supplementary Fig. 2. Profile of TEs involved in TE-G transcripts and ATE-G isoforms. **a)** Exonic positions of TEs identified in TE-G transcripts or ATE-G isoforms. Definitions for the features are displayed in the right panel. **b)** Contribution of TEs in TE-G transcripts or ATE-G isoforms to CDS, 5′/3′-UTRs. CDS, 5′/3′-UTRs in DRS-AtRTD3 and AtRTD3 were predicted using annotations from the AtRTD3 dataset. Examples of the counting of features are displayed in the bottom panels. **c)** Number of exonic, partial exonic, and intronic TEs found by ParasiTE in DRS-AtRTD3 and DRS-Araport11. See the Supplementary Note 1 for details on the criteria of the classes. Source data are provided as a Source Data file.

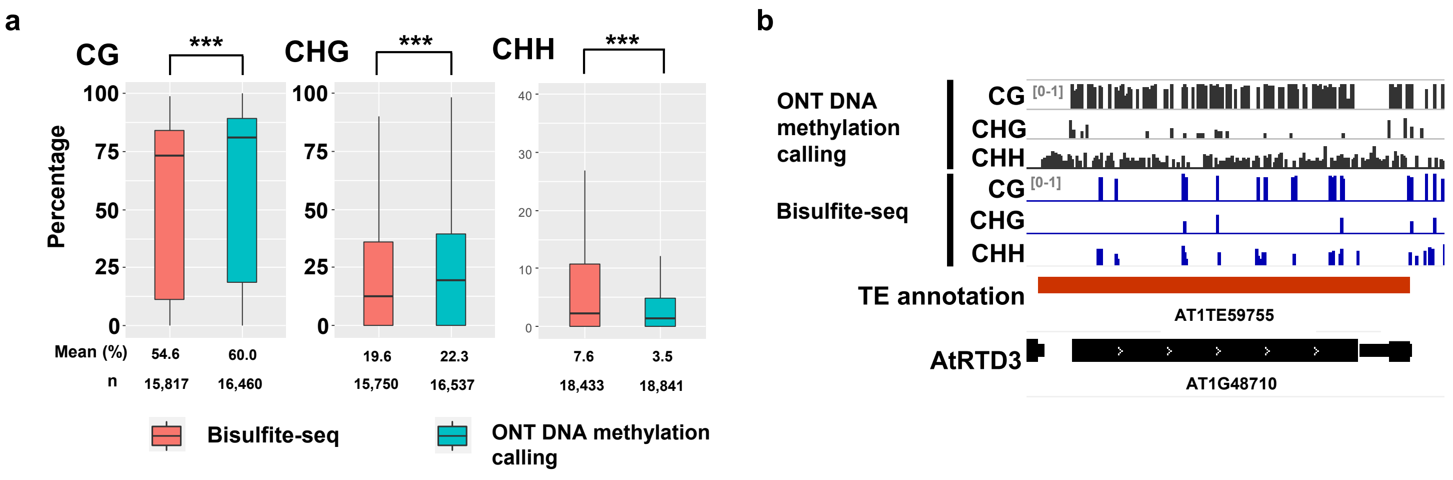

Supplementary Fig. 3. Comparison of methylation calling technologies at TE locus. **a)** Comparison of methylated CG, CHG, and CHH in TEs obtained by Bisulfite-seq[1] and ONT DNA methylation calling. ***, p<0.001 by the Mann–Whitney U test. The centerline represents the median. The borders of the boxplots are the first and third quartiles (Q1 and Q3). Whiskers represent data range, bounded to 1.5 * (Q3-Q1).
b) Genome browser tracks showing a comparison of DNA methylation calling obtained from Bisulfite-seq[1] and ONT DNA methylation calling at the LTR/*ATCOPIA78* (*ONSEN*) (AT1TE59755) locus. Source data are provided as a Source Data file.

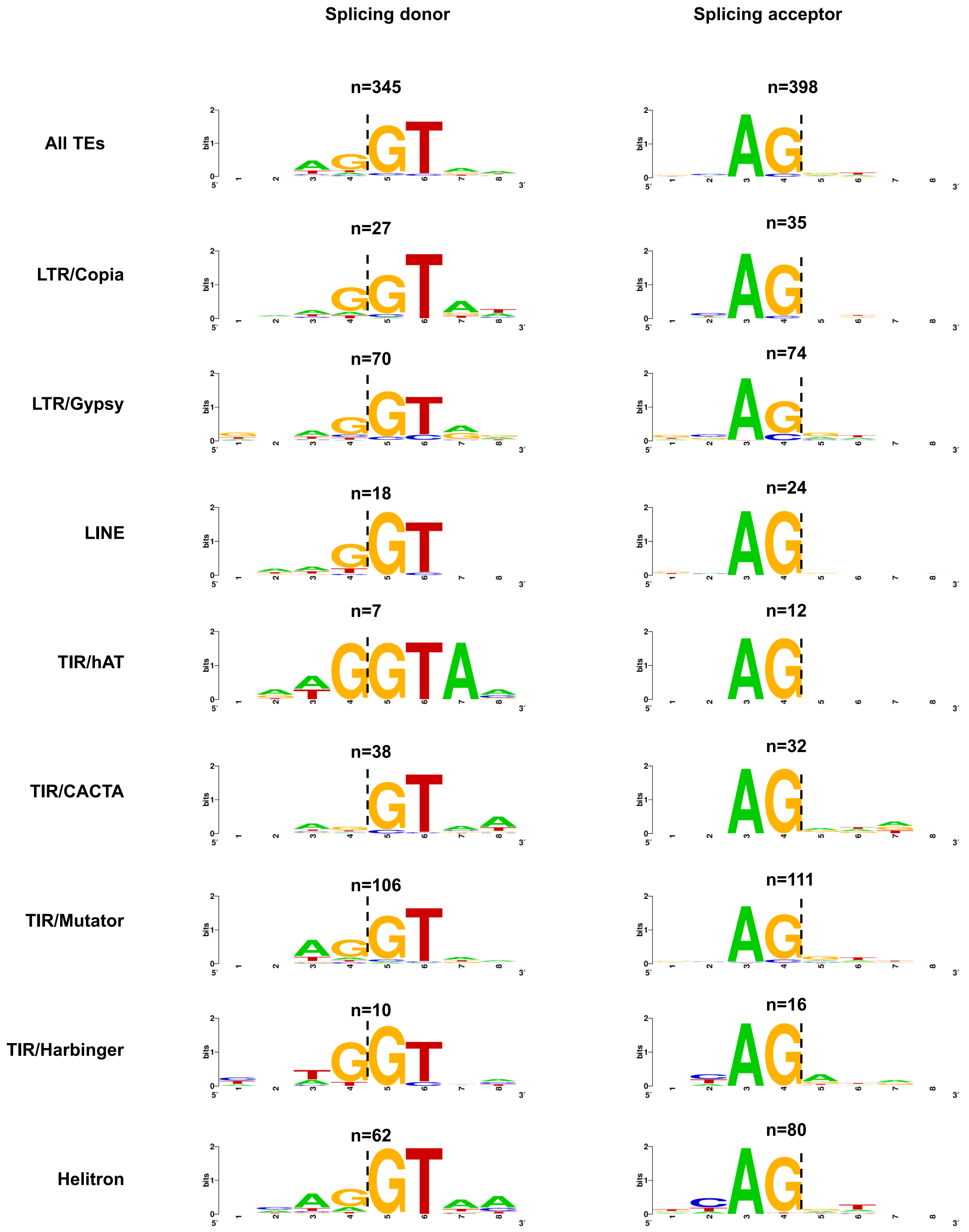

Supplementary Fig. 4. Enrichment of nucleotides at splicing donor and acceptor sites associated with TEs involved in TE-IR. The analysis was performed for all TEs as well as TE superfamilies associated with at least n=7 donor and acceptor splicing sites. Source data are provided as a Source Data file.

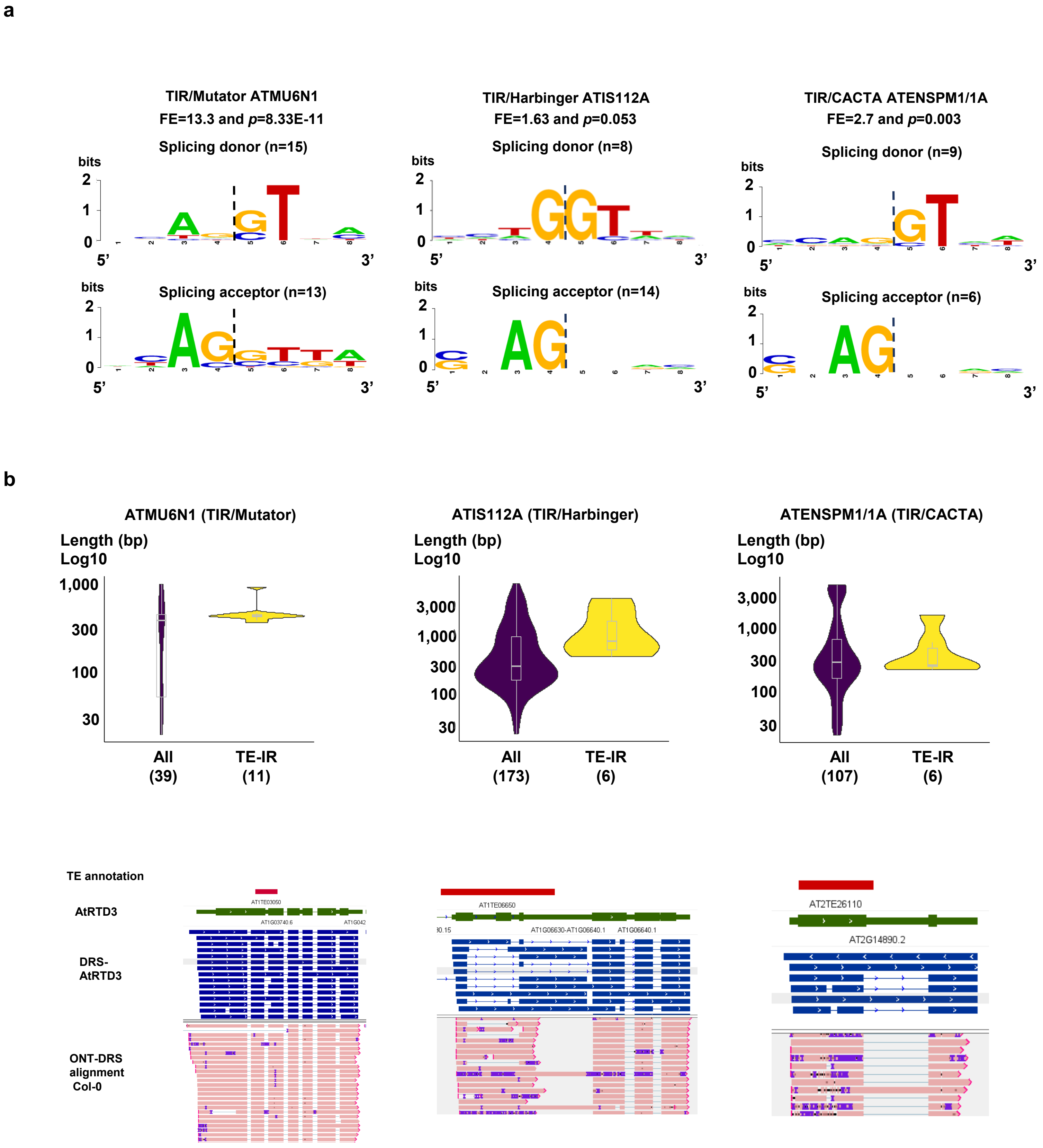

Supplementary Fig. 5. Profiles of TE families enriched in TE-IR events. **a)** Enrichment of nucleotides at splicing donor and splicing acceptor sites associated with TE families ATMU6N1, ATIS112A, and ATENSPM1/1A. Fold enrichment (FE) and *p*-values obtained by the hypergeometric test are indicated. **b)** Length of TEs belonging to TE families in a) associated with TE-IR events. Bottom panels are genome browser tracks showing DRS alignments of representative loci associated with TE-IR events of the TE families. The centerline represents the median. The borders of the boxplots are the first and third quartiles (Q1 and Q3). Whiskers represent data range, bounded to 1.5 * (Q3-Q1). Source data are provided as a Source Data file.

a

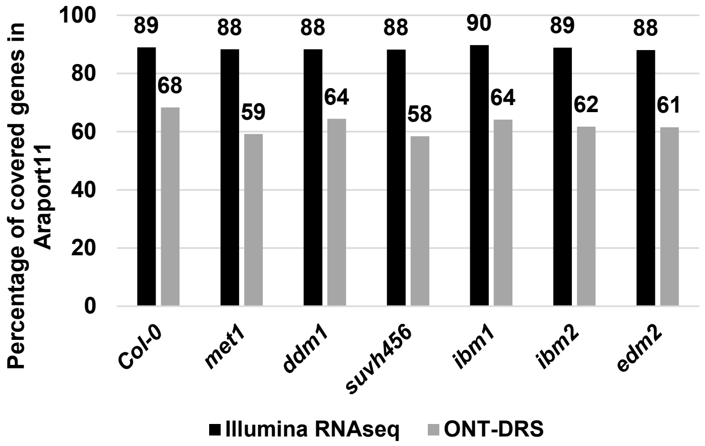

b

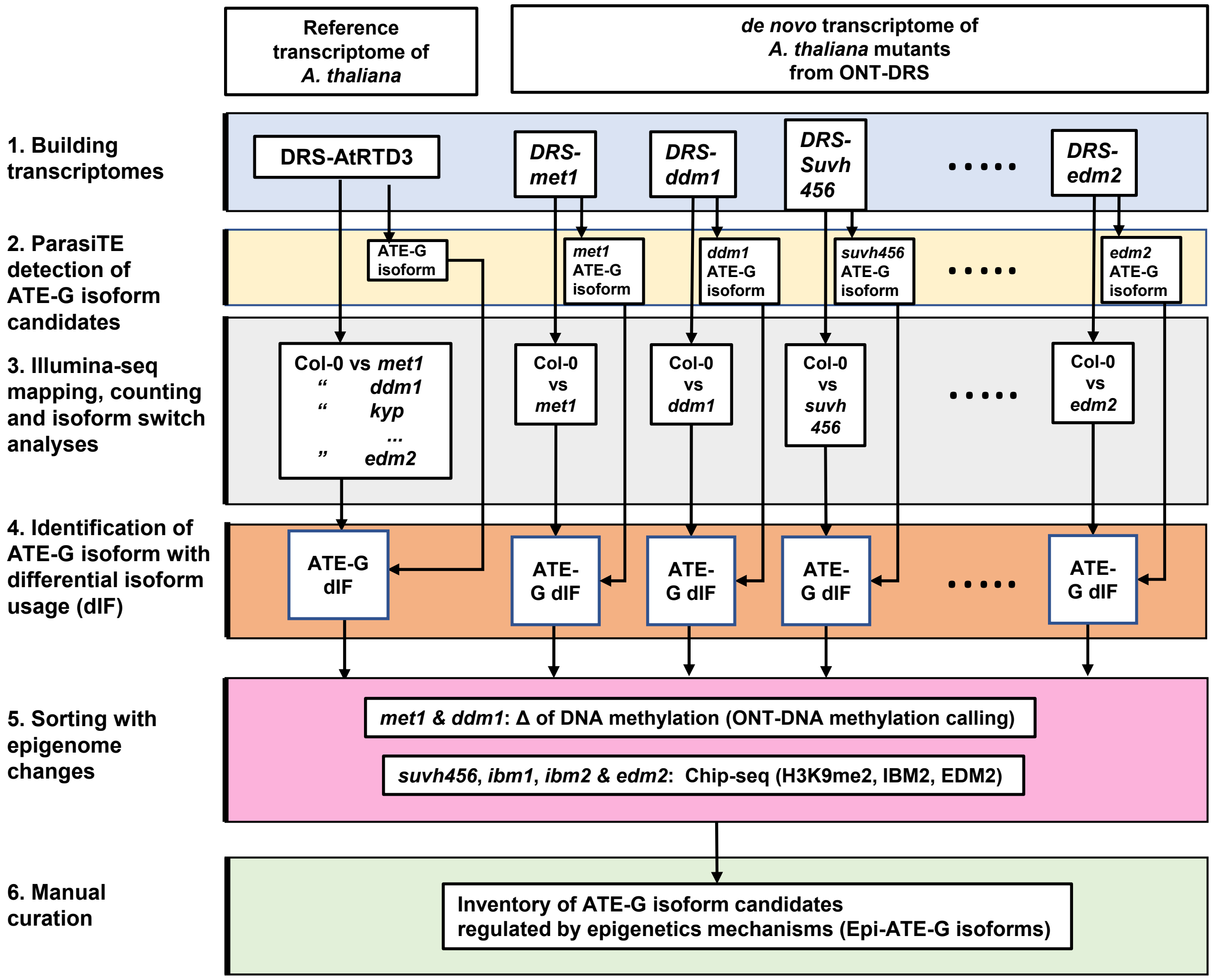

Supplementary Fig. 6. ONT-DRS sequencing of epigenetic mutants and identification of Epi-ATE-G isoforms. **a)** Coverage of Araport11 genes by at least 10 reads with ONT-DRS and Illumina RNA-seq in Col-0 and epigenetic mutants. **b)** Scheme of the methodology used to detect Epi-ATE-G isoforms: 1) DRS-AtRTD3 was obtained by merging the AtRTD3 transcriptome annotation and the new DRS-Col-0 transcriptome annotation. In parallel, *de novo* transcriptome assembly of epigenetic mutants was built from the ONT-DRS data; 2) ParasiTE detected ATE-G isoform candidates in the DRS-AtRTD3 and mutant-DRS datasets; 3) and 4) Illumina RNA-seq data of Col-0 and epigenetic mutants were aligned to the DRS-AtRTD3 transcriptome assembly. Next, ATE-G isoforms in DRS-AtRTD3 displaying significant isoform switch events were extracted. Following this, Illumina RNA-seq data of Col-0 and mutants were also aligned to the mutant-DRS datasets. Next, ATE-G isoforms in mutant-DRS associated with significant isoform switch events were retrieved; 5) The direct effects of epigenome changes on Epi-ATE-G isoforms were verified. Epi-ATE-G isoforms associated with DNA methylation changes in *met1* and *ddm1*, H3K9me2 ChIP-seq peaks in Col-0 for *suvh456,* H3K9me2 ChIP-seq peaks in Col-0 and *ibm1* for *ibm1*, and IBM2/EDM2 ChIP-seq peaks for *ibm2* and *edm2* were extracted. Source data are provided as a Source Data file.

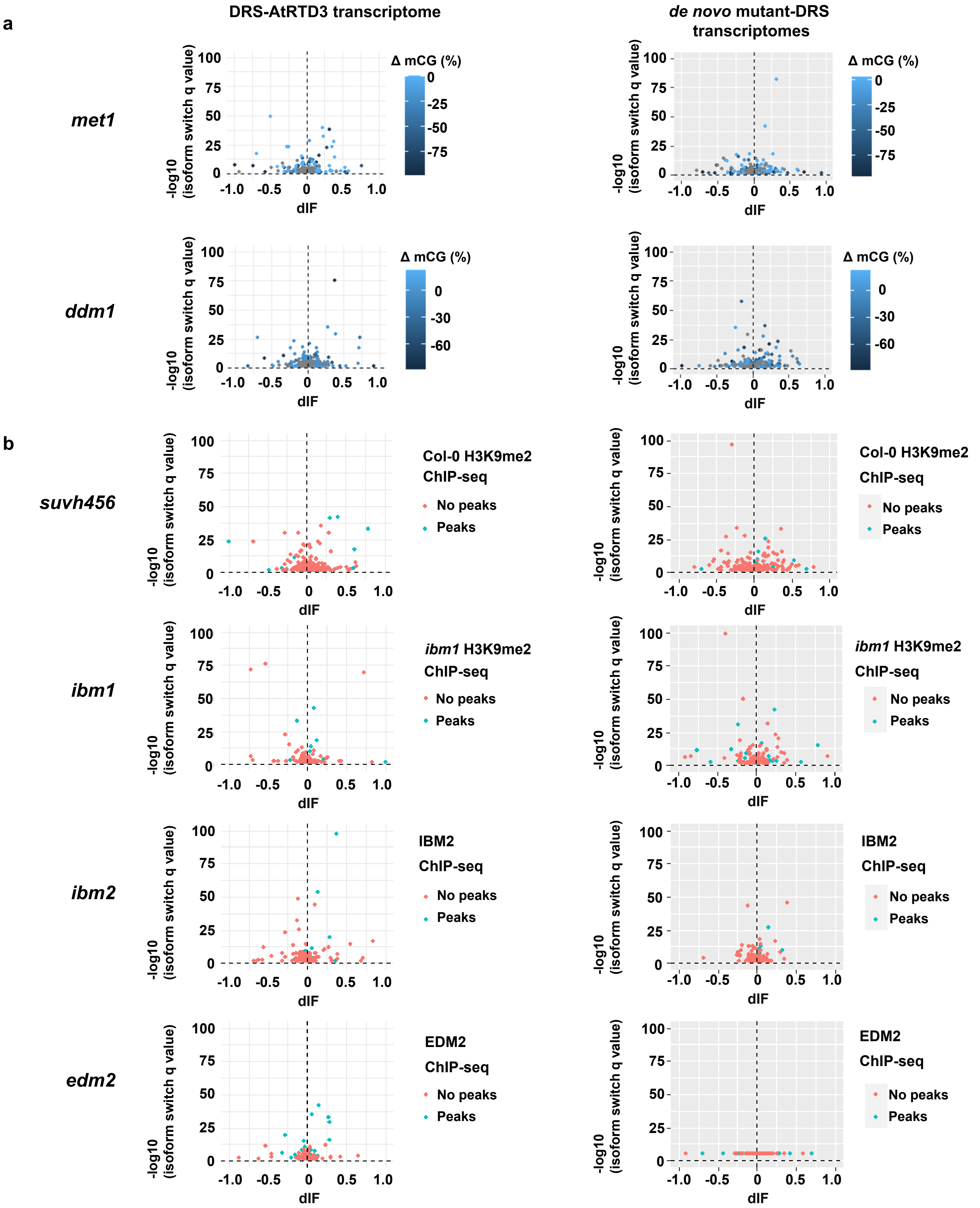

Supplementary Fig. 7. Volcano plots showing differential isoform usage (dIF) values for mutants compared to Col-0. **a)** dIF of each ATE-G isoform (dot) with a significant isoform switching (|dIF|> 0.01, q<0.05) in *met1* and *ddm1* compared to Col-0 using DRS-AtRTD3 (left) or *mutant*-DRS transcriptome (right) as references. Changes in CG methylation in associated TE is indicated by a light-dark blue in each dot. **b)** dIF of each ATE-G isoform (dot) with a significant isoform switching (|dIF|> 0.01, q<0.05) in *suvh456*, *ibm1*, *ibm2* and *edm2* compared to Col-0 using DRS-AtRTD3 (left) or *mutant*-DRS transcriptome (right) as references. Association of H3K9me2 ChIP-seq peaks with TE in Col-0 is indicated (light blue) for *suvh456* and *ibm1*. Association of IBM2 ChIP-seq peaks and EDM2 ChIP-seq peaks with TE are indicated (light blue) for *ibm2* and *edm2*, respectively. Source data are provided as a Source Data file.

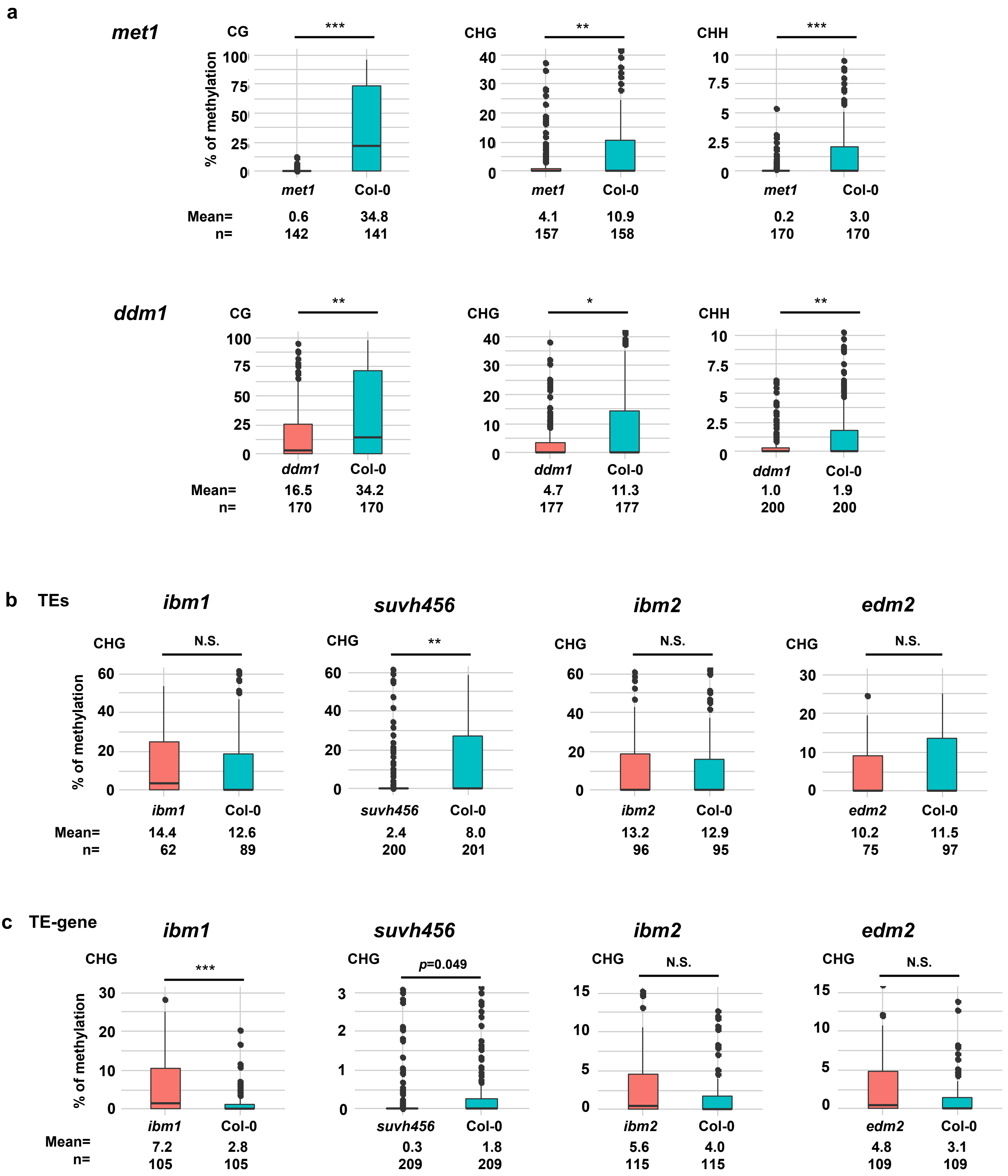

Supplementary Fig. 8. Changes in DNA methylation of TEs associated in ATE-G isoforms with significant isoform switching in mutants compared to Col-0 (|dIF|> 0.01, q<0.05). **a)** Changes in DNA methylation of TEs associated in ATE-G isoforms with significant isoform switching detected in *met1* and *ddm1* compared to Col-0 using *met1*-DRS background and *ddm1*-DRS background as references, respectively (Supplementary Data 6). **b)** Changes in DNA CHG methylation of TEs associated in ATE-G isoforms with significant isoform switching detected in *suvh456*, *ibm1*, *ibm2* and *edm2* compared to Col-0 using DRS-AtRTD3 as a reference (Supplementary Data 5).
c) Changes in DNA CHG methylation of TE-gene loci associated in ATE-G isoforms with significant isoform switching detected in *suvh456*, *ibm1*, *ibm2* and *edm2* compared to Col-0 using DRS-AtRTD3 as a reference. *; *p*<0.05, **; *p*<0.01, ***; *p*<0.001, N.S.; Not Significant, by the Mann–Whitney *U* test. The centerline represents the median. The borders of the boxplots are the first and third quartiles (Q1 and Q3). Whiskers represent data range, bounded to 1.5 * (Q3-Q1). Points outside this range are represented individually by black circles. Source data are provided as a Source Data file.

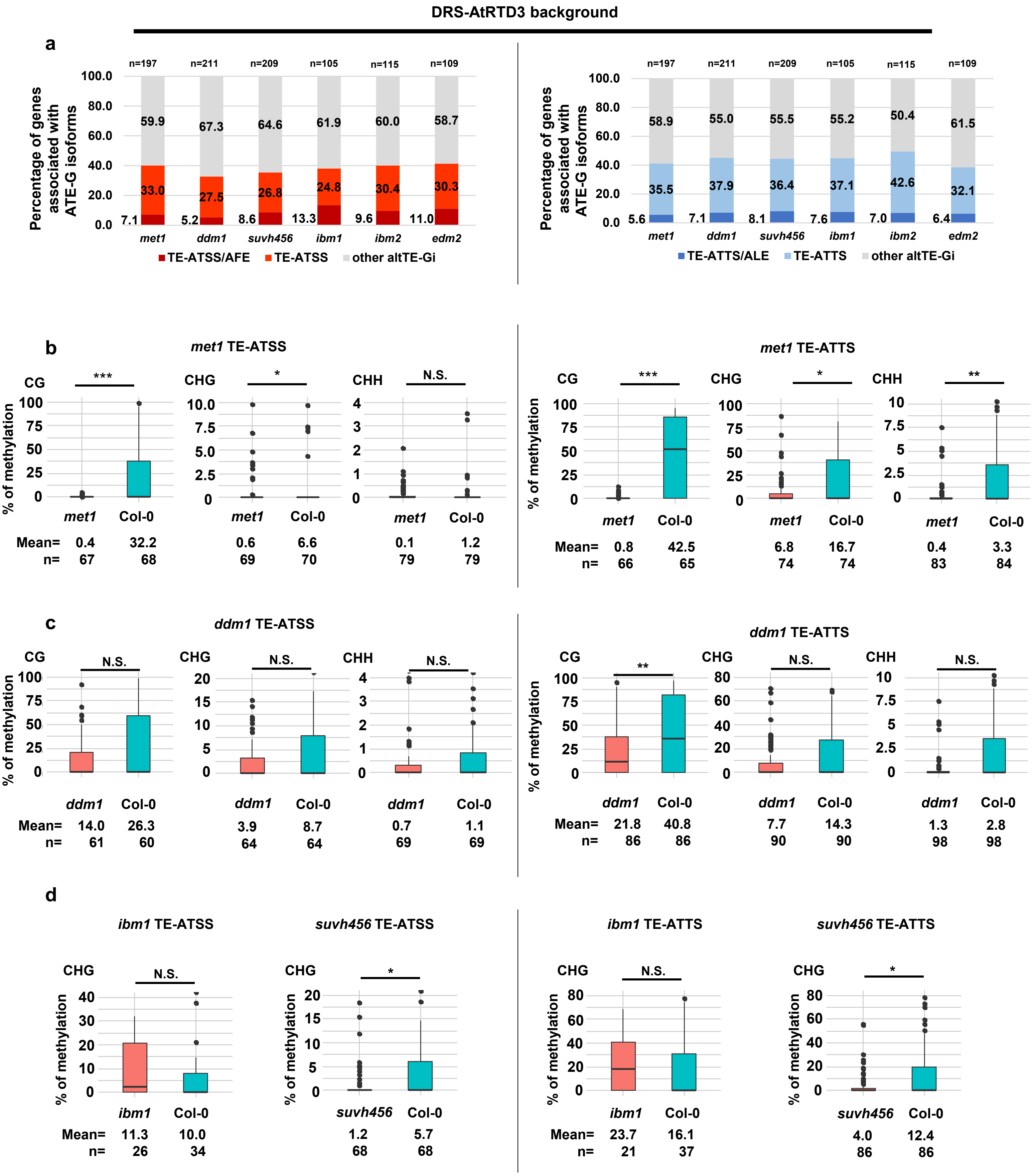

Supplementary Fig. 9. TE-ATSS and TE-ATTS with significant isoform switching in mutants compared to Col-0 (|dIF|> 0.01, q<0.05) detected with DRS-AtRTD3 (Supplementary Data 5). **a)** Contribution of TE-ATSS (left) and TE-ATTS (right) among ATE-G isoforms with significant isoform switching compared to Col-0 (|dIF|> 0.01, q<0.05) detected in mutants with DRS-AtRTD3. **b)** DNA methylation of TEs associates with TE-ATSS (left) and TE-ATTS (right) with significant isoform switching in *met1* compared to Col-0. **c)** DNA methylation of TEs associates with TE-ATSS (left) and TE-ATTS (right) with significant isoform switching in *ddm1* compared to Col-0. **d)** DNA methylation in TEs associates with TE-ATSS (left) and TE-ATTS (right) with significant isoform switching in *ibm1* or *suvh456* compared to Col-0. For b), c) and d), the centerline represents the median; the borders of the boxplots are the first and third quartiles (Q1 and Q3). Whiskers represent data range, bounded to 1.5 * (Q3-Q1). Points outside this range are represented individually by black circles. Source data are provided as a Source Data file.

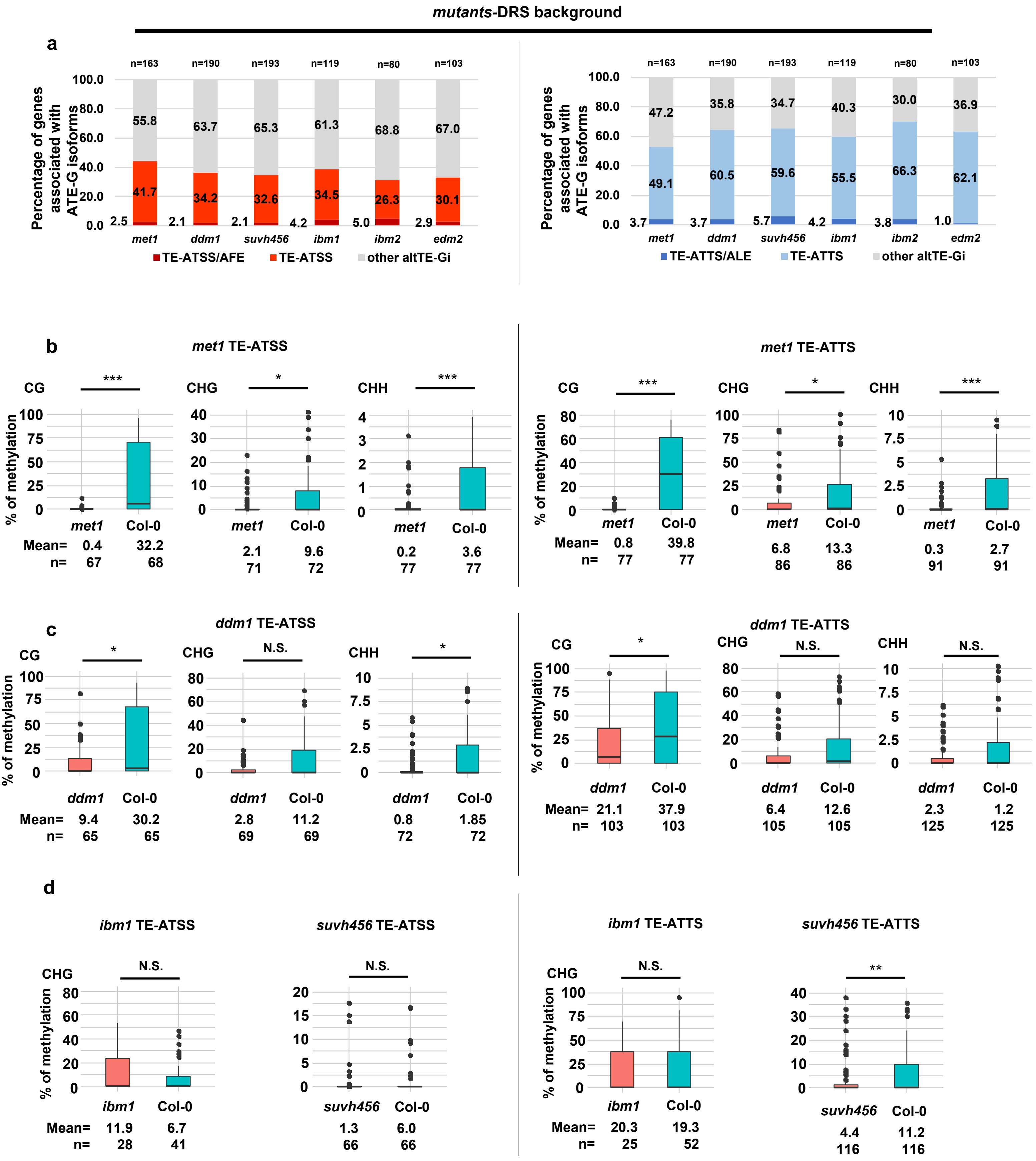

Supplementary Fig. 10. TE-ATSS and TE-ATTS with significant isoform switching in mutants compared to Col-0 (|dIF|> 0.01, q<0.05) detected with the mutant-DRS transcriptomes (Supplementary Data 6). **a)** Contribution of TE-ATSS (left) and TE-ATTS (right) among ATE-G isoforms with significant isoform switching (|dIF|> 0.01, q<0.05) detected in mutants with the mutant DRS reference. **b)** DNA methylation of TEs associates with TE-ATSS (left) and TE-ATTS (right) with significant isoform switching in *met1* compared to Col-0. **c)** DNA methylation of TEs associates with TE-ATSS (left) and TE-ATTS (right) with significant isoform switching in *ddm1* compared to Col-0. **d)** DNA methylation of TEs associates with TE-ATSS (left) and TE-ATTS (right) with significant isoform switching in *ibm1* or *suvh456* compared to Col-0. For b), c) and d), the centerline represents the median, the borders of the boxplots are the first and third quartiles (Q1 and Q3). Whiskers represent data range, bounded to 1.5 * (Q3-Q1). Points outside this range are represented individually by black circles. Source data are provided as a Source Data file.

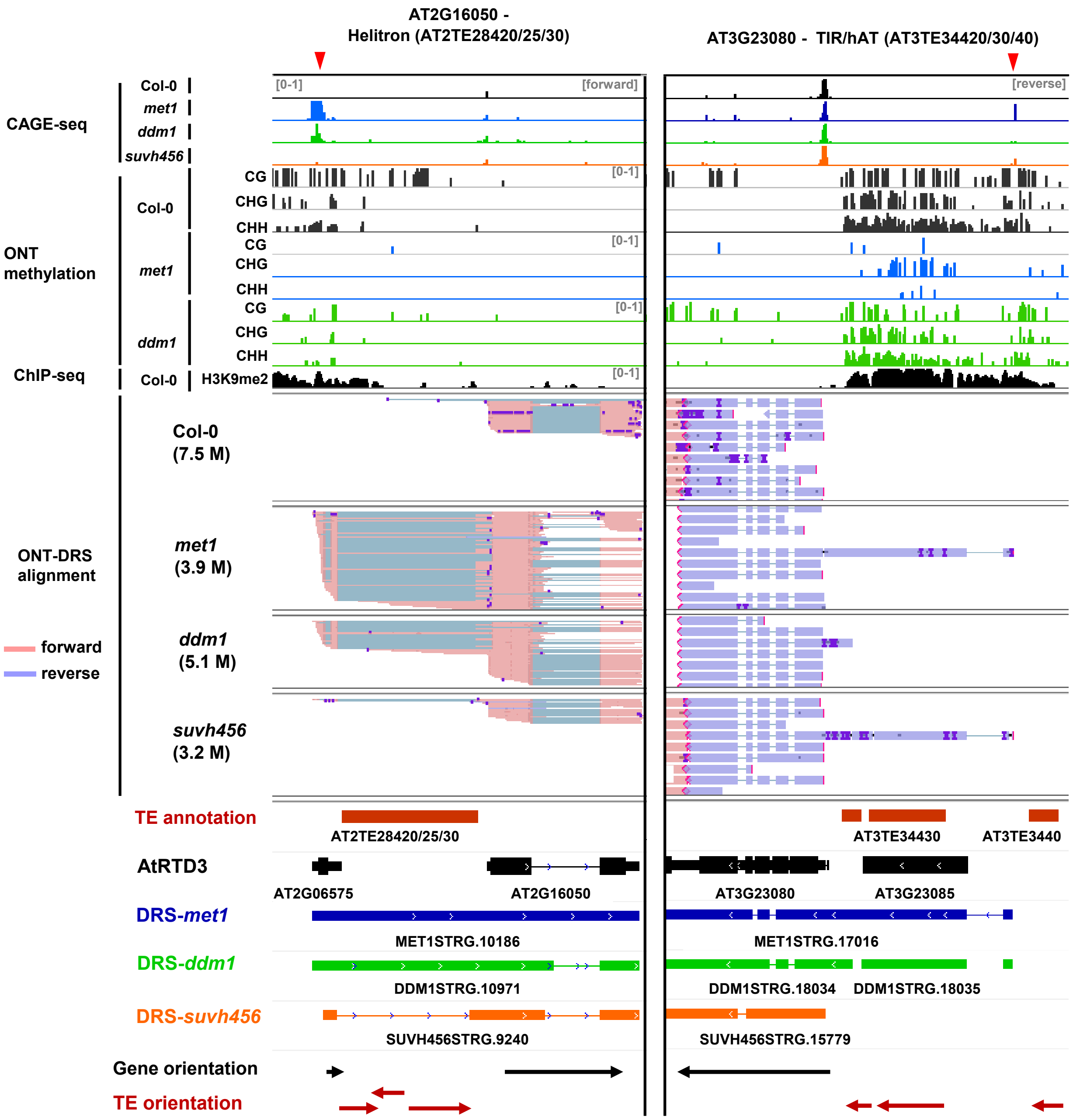

Supplementary Fig. 11. Epigenetic regulation of TE-ATSS. Representative genome loci showing Epi-ATE-G isoforms with TE-ATSS events. Tracks (from top to bottom): CAGE-seq (reads per million; 0–1. Only forward or reverse strands are shown); methylation level of each mutant in CG, CHG, and CHH contexts (0–100%); Col-0 ChIP-seq of H3K9me2 (reads per million; 0–1); DRS read alignments of Col-0 and indicated mutants; TE and AtRTD3 transcript annotations, *de novo* assembly of transcripts in mutants, and the orientation of genes and TEs. Red arrows indicate cryptic TSSs detected in the epigenetic mutants.

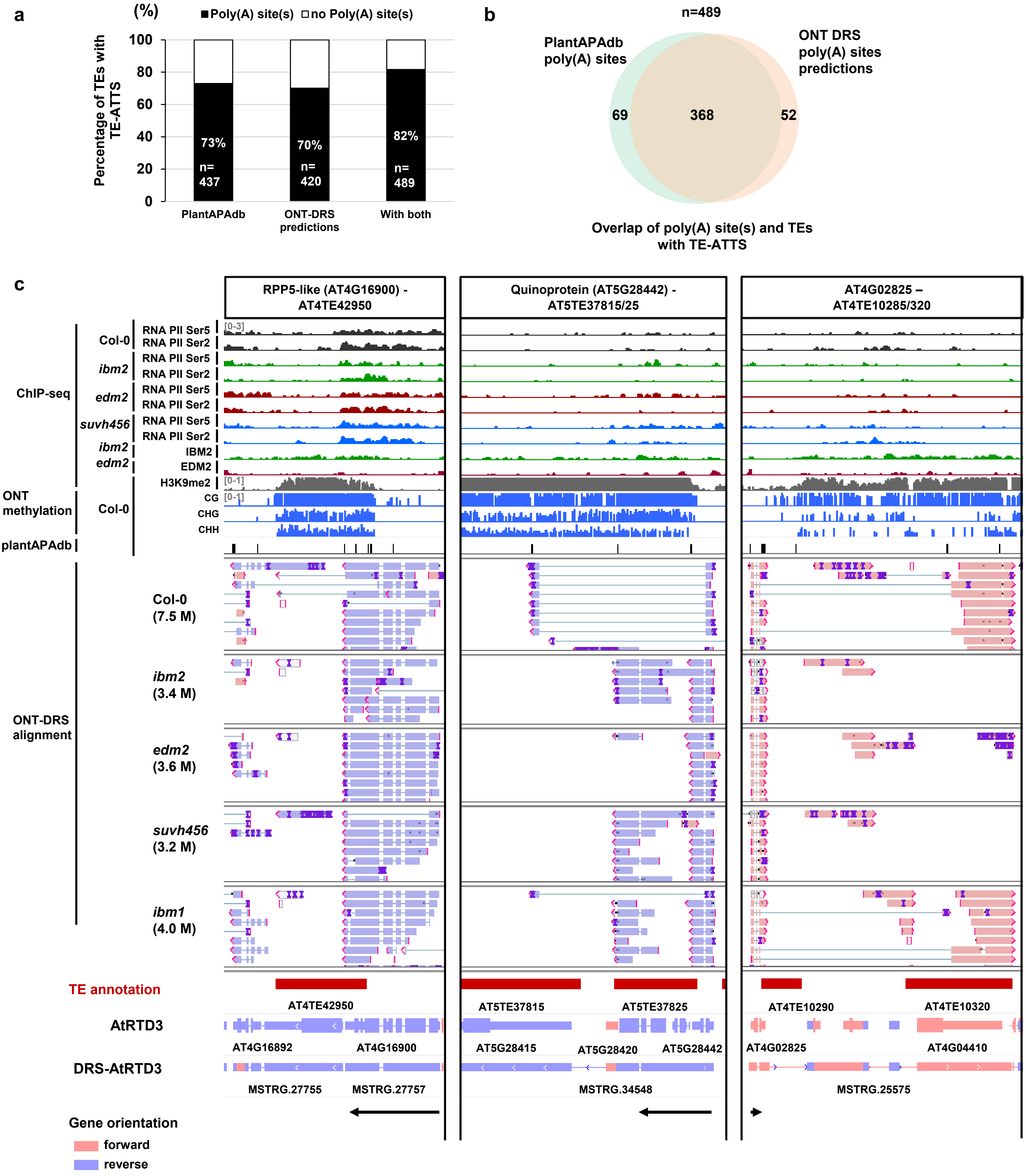

Supplementary Fig. 12. TEs with TE-ATTS events that overlap with poly(A) sites and epigenetic regulation of TE-ATTS. **a)** TEs with TE-ATTS events that overlap with poly(A) sites in the PlantAPA database or detected in the DRS data. **b)** Overlap of poly(A) sites with TEs associated with ATTS. **c)** Epigenetic regulation of TE-ATTS. Representative genome loci showing Epi-ATE-G isoforms with TE-ATTS events. Tracks (from top to bottom): ChIP-seq data for RNA Pol II phosphorylated at Ser5/Ser2 in CTD repeats (bins per million); ChIP-seq data for IBM2 and EDM2 localization (bin per million); Col-0 ChIP-seq of H3K9me2 (reads per million); methylation levels of Col-0 in CG, CHG, and CHH contexts (0–100%); poly(A) sites obtained from the PlantAPA database; DRS read alignments of Col-0 and indicated mutants; TE and transcript annotations of AtRTD3 and DRS-AtRTD3 in this study and the orientation of genes. Source data are provided as a Source Data file.

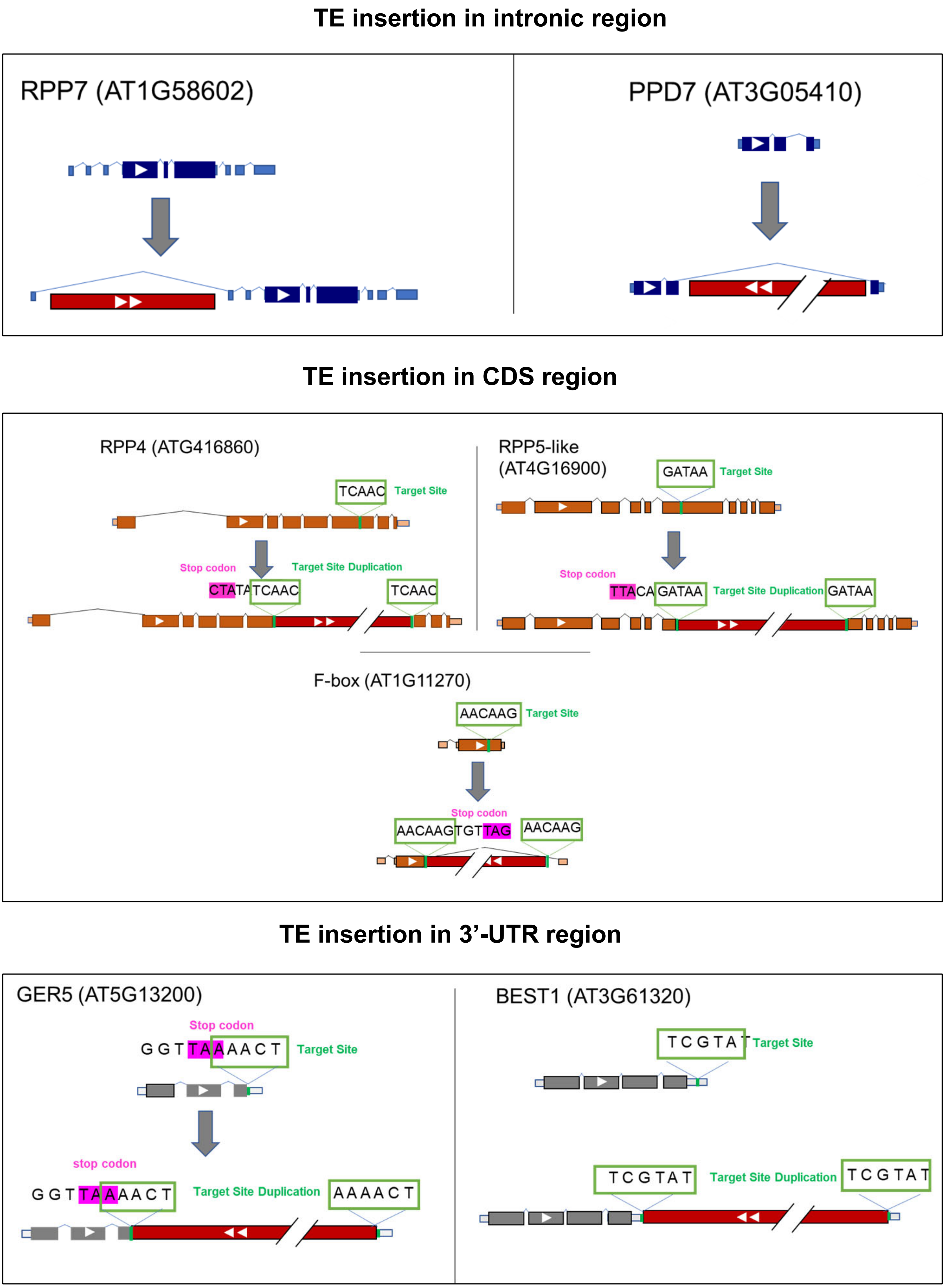

Supplementary Fig. 13. Insertion of TEs in intragenic regions (intron, CDS/exon, and 3′-UTR) of genes analyzed in this study. Target site duplications are indicated. For *PPD7*, *GER5*, and *BEST1*, the Ler-0 genome sequence was used as an ancestral reference ecotype before TE insertion.

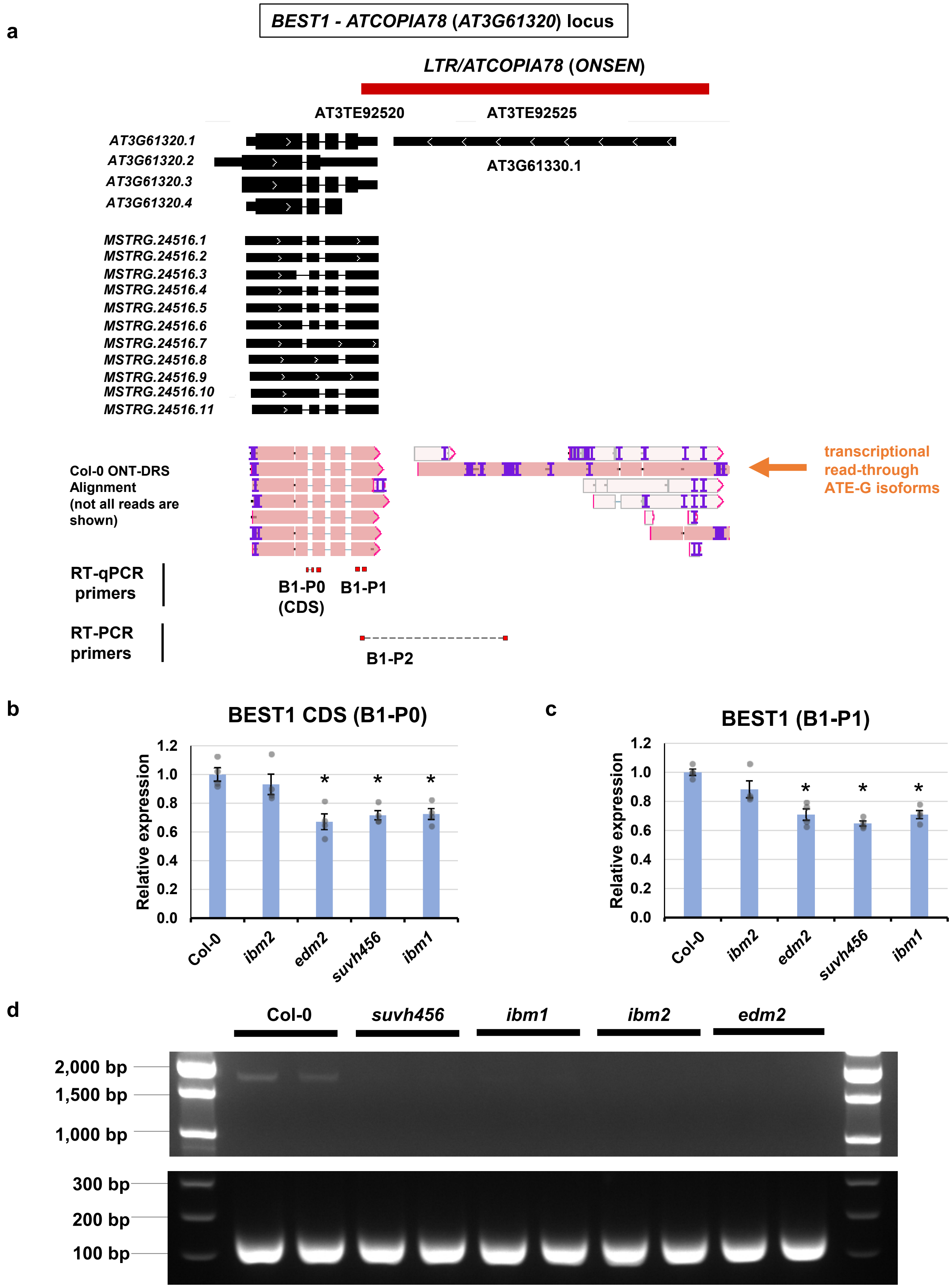

Supplementary Fig. 14. *BEST1-ATCOPIA78* (*ONSEN*) (*AT3G61320*) locus. **a)** Genome browser tracks showing the complete DRS-AtRTD3 transcript annotation. The Col-0 ONT-DRS alignment is also shown with a transcriptional read-through ATE-G isoform detected in the Col-0-DRS data (orange arrow). Positions of primers used for RT-qPCR in b and c and RT-PCR in d are indicated. **b), c)** Relative expression of *BEST1* CDS and ATE-G isoforms in Col-0 and the epigenetic mutants. Bars represent the means of four biological replicates ± SEM. *, $p < 0.05$ by *t*-test. **d)** Agarose gel electrophoresis of RT-PCR amplicons with primers targeting the predicted transcriptional read-through of *BEST1*. *ACT2* (*AT3G18780*) was used as a positive control. Source data are provided as a Source Data file.

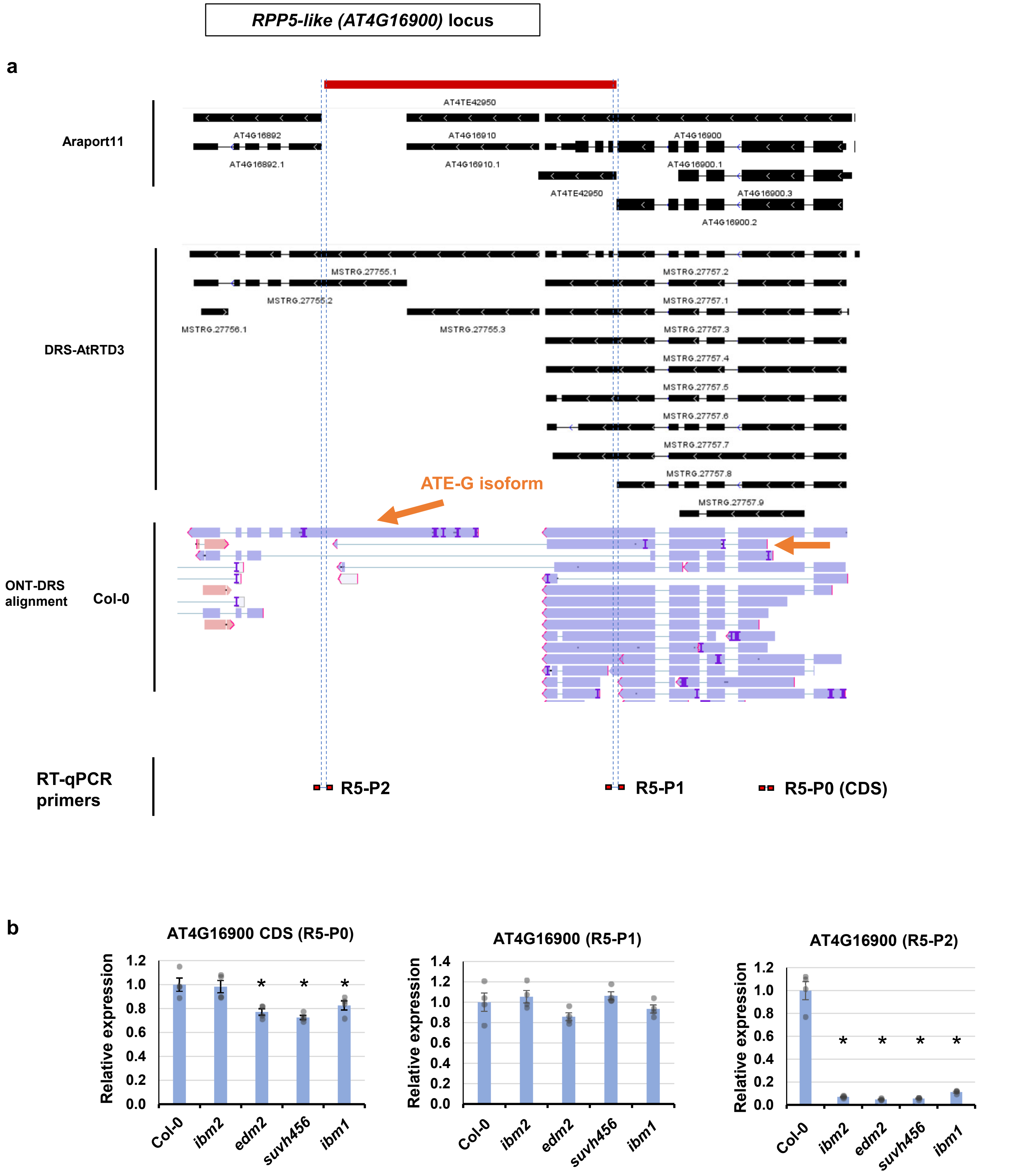

Supplementary Fig. 15. The *RPP5-like-AT4TE42950* (*AT4G16900*) locus. **a)** Genome browser tracks showing the complete DRS-AtRTD3 transcript annotation, DRS reads, and primer positions for RT-qPCR in b. Arrows indicate ATE-G isoforms covering the downstream *AT4G16892* locus. **b)** Relative expression of the *RPP5-like* CDS and ATE-G isoforms in Col-0 and epigenetic mutants. Target-specific primers for RT-qPCR are illustrated above. Bars represent the means of four biological replicates ± SEM. *, $p < 0.05$ by *t*-test. Source data are provided as a Source Data file.

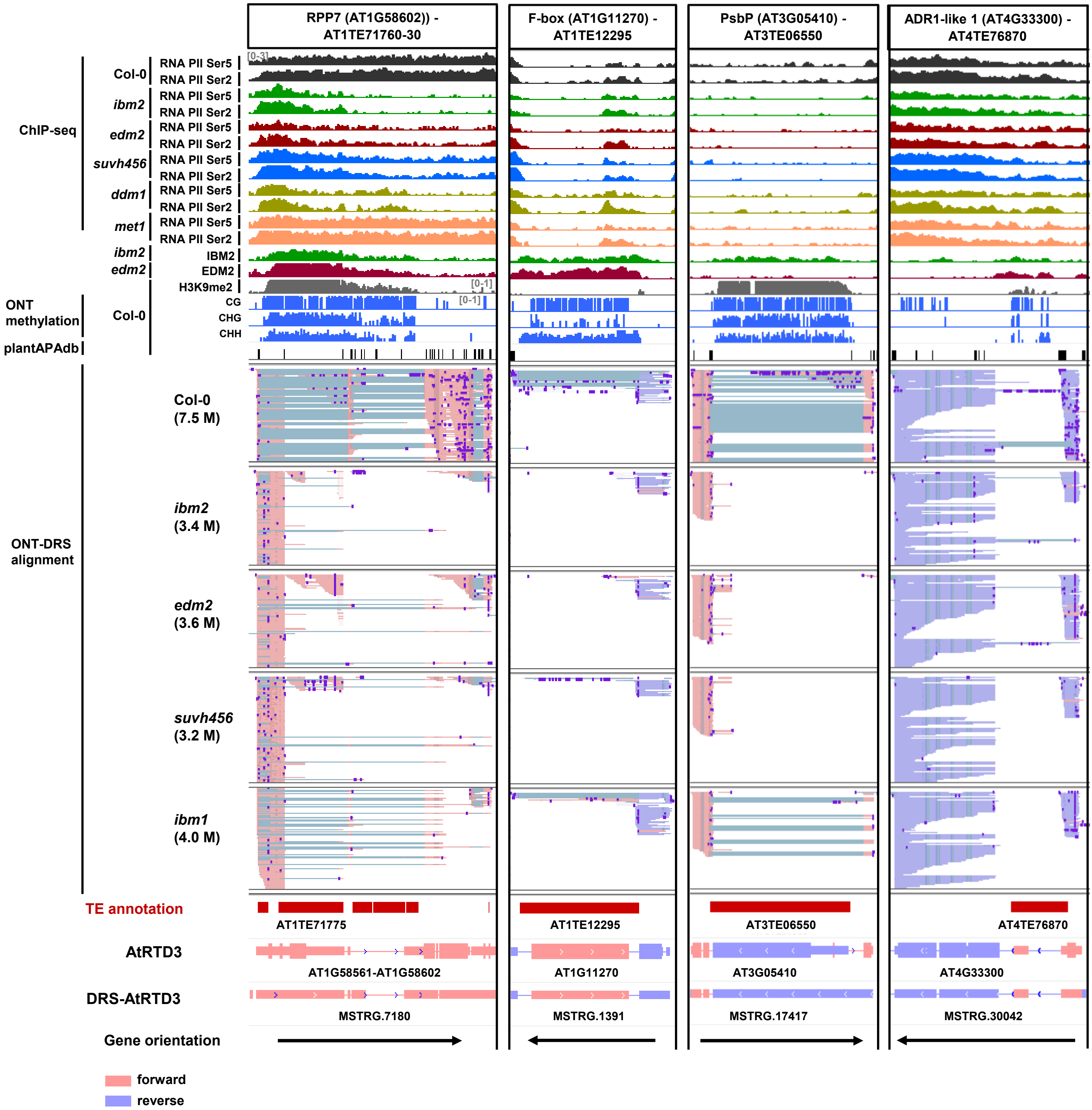

Supplementary Fig. 16. Epigenetic regulation of intronic TEs and ATE-G isoform production. Representative genome loci showing Epi-ATE-G isoform production with TE-ATTS events. Tracks (from top to bottom): ChIP-seq data for RNA Pol II phosphorylated at Ser5/Ser2 in CTD repeats (bins per million); ChIP-seq data for IBM2 and EDM2 localization (bins per million); Col-0 ChIP-seq of H3K9me2 (bins per million); methylation level of Col-0 in CG, CHG, and CHH contexts (0–1); poly(A) sites obtained from the PlantAPA database; DRS read alignments of Col-0 and indicated mutants; TE and transcript annotations of AtRTD3 and DRS-AtRTD3 in this study and the orientation of genes.

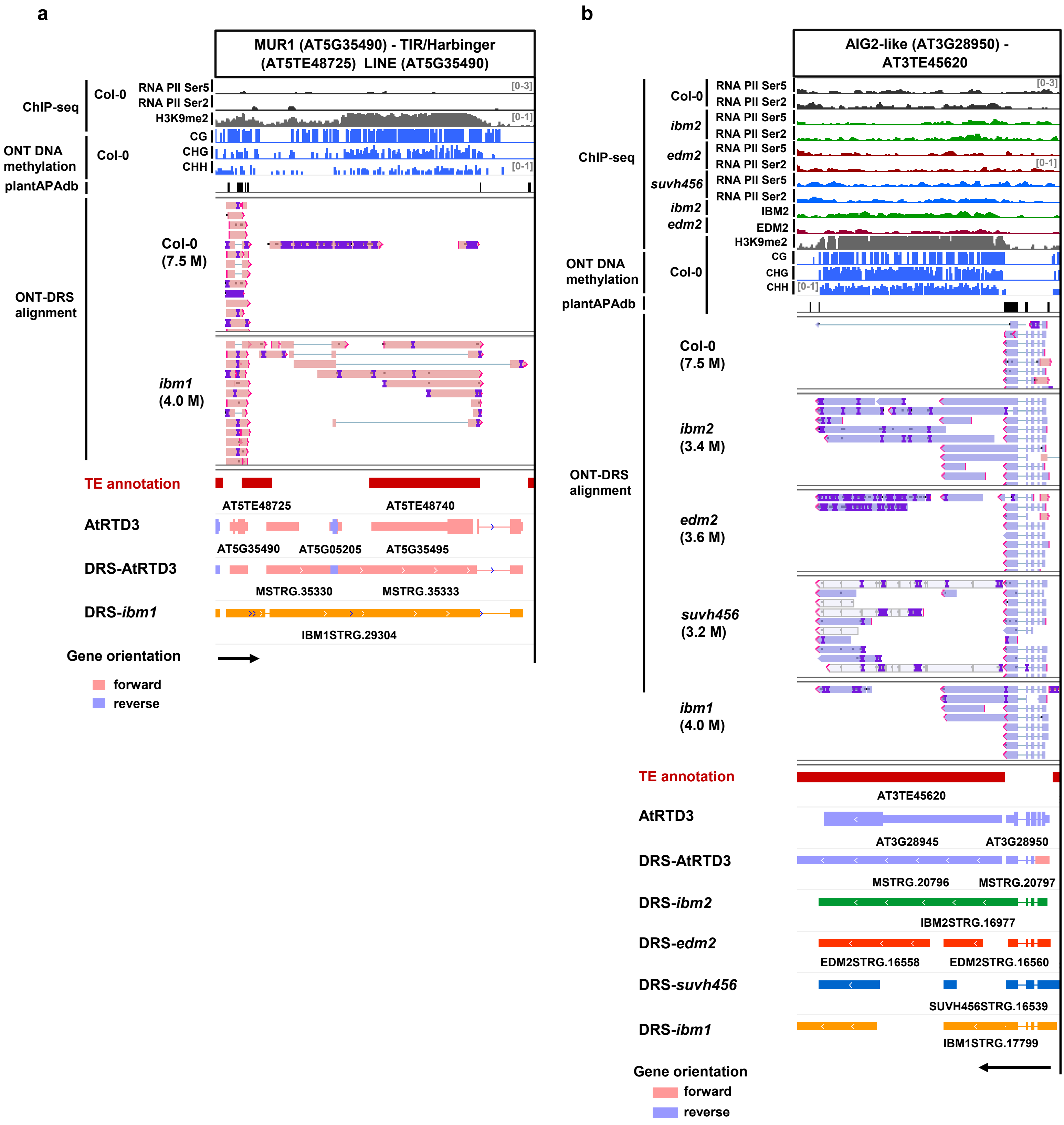

Supplementary Fig. 17. Epigenetic regulation of TE-ATTS events at *MUR1* (AT5G35490) and *AIG2-like* (AT3G28950) gene loci. **a)** *MUR1* (*AT5G35490*) and **b)** *AIG2-like* (*AT3G28950*) gene loci showing Epi-ATE-G isoform production with TE-ATTS events, which were particularly enhanced in the mutant background. Tracks (from top to bottom): ChIP-seq data for RNA Pol II phosphorylated at Ser5/Ser2 in CTD repeats (bin per million); Col-0 ChIP-seq of H3K9me2 (reads per million); methylation levels of Col-0 in CG, CHG, and CHH contexts (0–100%); poly(A) sites obtained from the PlantAPA database; DRS read alignments of Col-0 and indicated mutants; TE and transcript annotations of AtRTD3, DRS-AtRTD3, and mutant-DRS in this study and the orientation of genes.

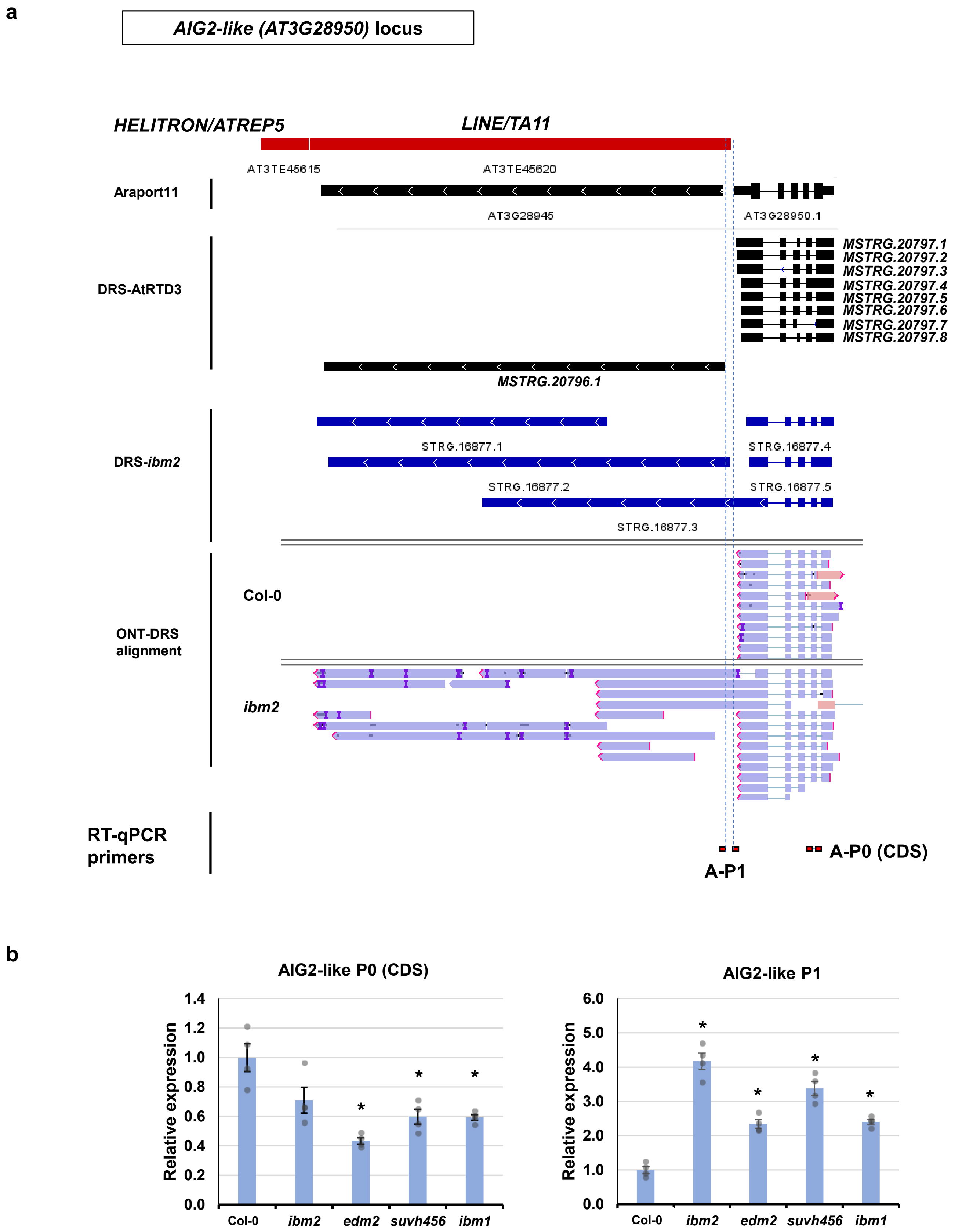

Supplementary Fig. 18. *AIG2-like-TA11* (*AT3G28950*) locus. **a)** Genome browser tracks showing the complete DRS-AtRTD3 transcript annotation, DRS reads, and primer positions for RT-qPCR in b. **b)** Relative expression of the *AIG2-like* CDS and ATE-G isoforms in Col-0 and the epigenetic mutants. Bars represent the means of four biological replicates ± SEM. *, $p < 0.05$ by *t*-test. Source data are provided as a Source Data file.

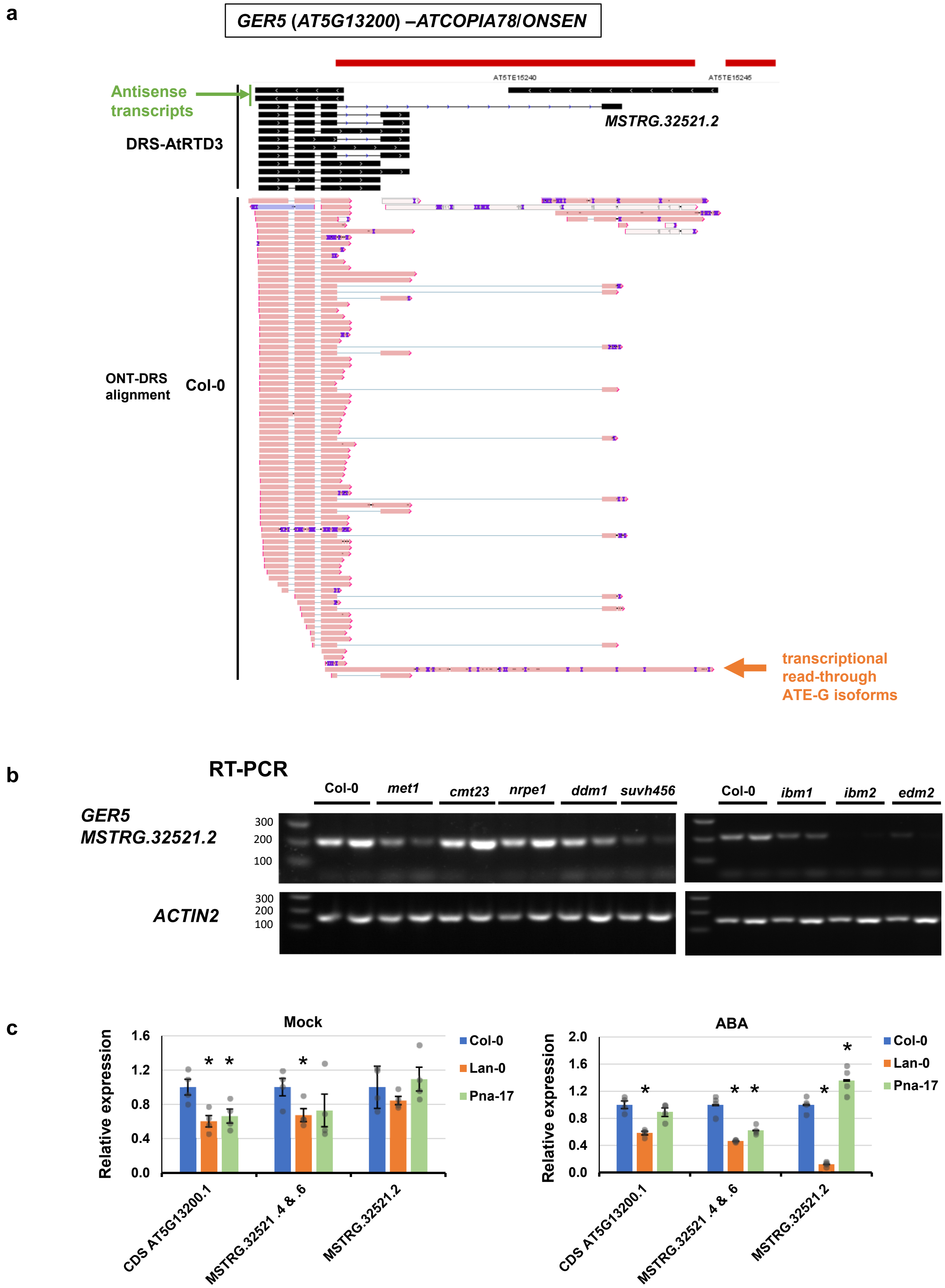

Supplementary Fig. 19. The *GER5-ATCOPIA78* (*ONSEN*) (*AT5G13200*) locus. **a)** Genome browser tracks showing the complete DRS-AtRTD3 transcript annotation and Col-0 DRS reads. The green arrow indicates an antisense transcript of *GER5* from the LTR sequence of *ONSEN*. A transcriptional read-through ATE-G isoform was also detected in the Col-0-DRS data (orange arrow). **b)** RT-PCR of ATE-G isoform (*MSTRG.32521.2*) in Col-0 and the epigenetic mutants. *ACTIN2* (*AT3G18780*) was used as a control. **c)** Relative expression of *GER5* CDS (*AT5G13200.1*) and ATE-G isoforms (*MSTRG.3521.2*, *4*, *6*) under mock and ABA stress conditions in ecotypes with the *ATCOPIA78/ONSEN* insertion (Col-0, Lan-0, and Pna-17). Expression levels in Col-0 were set as 1. Bars represent the means of four biological replicates ± SEM. *, $p < 0.05$ by *t*-test. Source data are provided as a Source Data file.

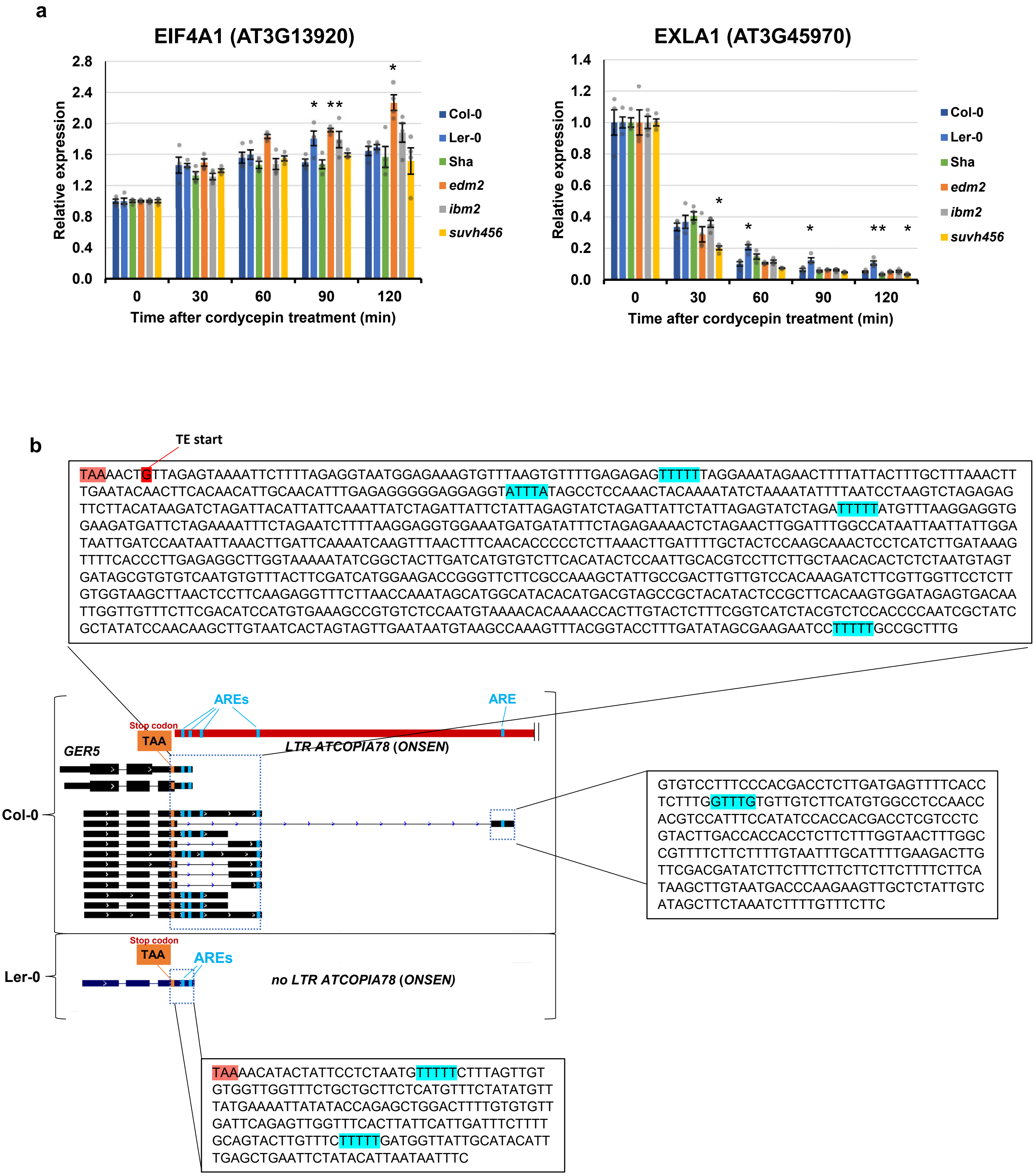

Supplementary Fig. 20. RNA stability analysis. **a)** Relative expression of eukaryotic initiation factor-4A (*EIF4A1, AT3G13920*) and EXPANSIN-LIKE A1 (*EXLA1, AT3G45970*) at time 0, 30, 60, 90, and 120 min after cordycepin treatment for Col-0, Ler-0, Sha, *ibm2*, *edm2*, and *suvh456*. Expression levels at 0 min were set as 1. Bars represent the means of four biological replicates ± SEM. *, $p < 0.05$ by *t*-test. *EIF4A1* and *EXLA1* were examined as control transcripts for high and low mRNA stability, respectively. **b)** The *ATCOPIA78/ONSEN* sequence in *GER5* locus provides an AU-rich element (indicated as ARE) in ATE-G isoform. Ler-0 with no *ATCOPIA78/ONSEN* insertion is also shown. Source data are provided as a Source Data file.

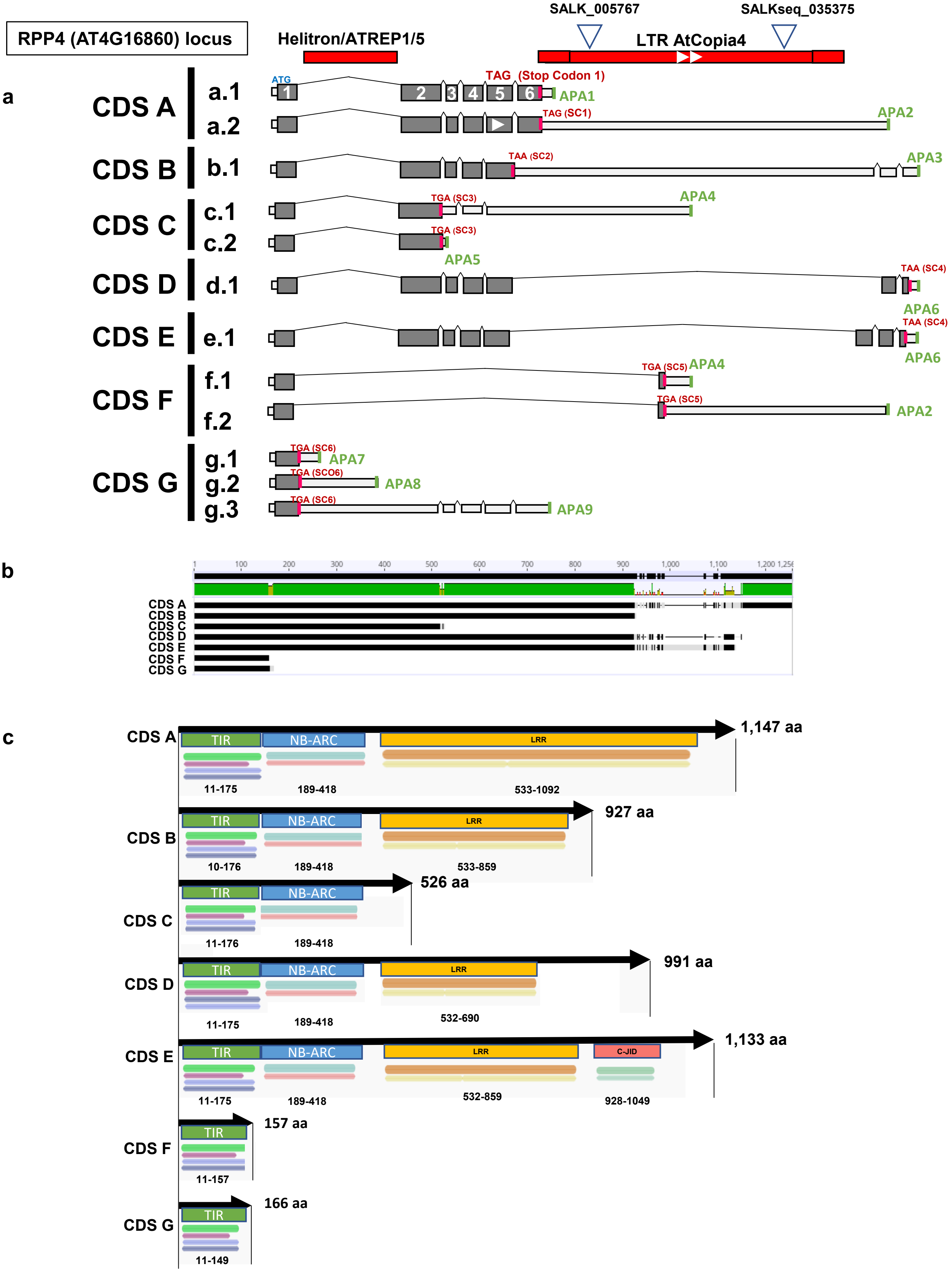

Supplementary Fig. 21. Prediction of protein CDS in the *RPP4* locus based on the ONT-DRS transcript data. **a)** Prediction of CDSs encoded by selected *RPP4* transcript isoforms predicted by DRS-AtRTD3 and manually retrieved DRS-ONT read alignments in Supplementary Fig. 23. Predicted top codons (SCs) and alternative poly(A) sites (APA) are also represented. **b)** Multiple amino acid alignments of predicted *RPP4* CDS (in the longest isoforms of CDS A–G) show that the *RPP4* locus encodes for at least seven protein isoforms. **c)** Prediction of protein domains in the transcript isoforms (CDS in the longest isoforms of CDS A–G).

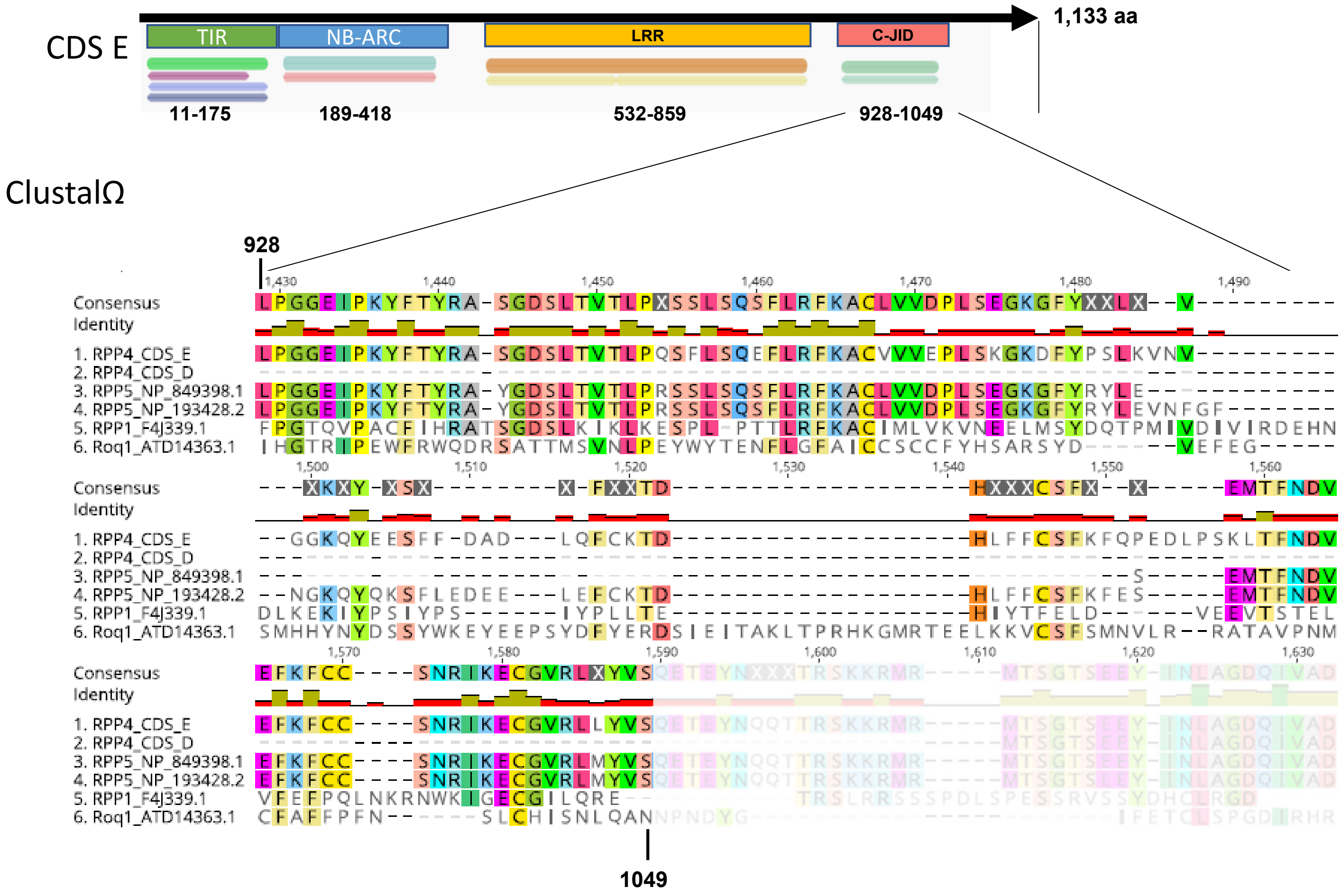

Supplementary Fig. 22. *RPP4* CDS “E” in Supplementary Fig. 21c is predicted to encode for a C-JID domain in the original protein sequence. Multiple alignments of the amino acids predicted to be encoded by “RPP4 CDS E”, “RPP4 CDS D” (no C-JID predicted), RPP5 proteins variants (ID: NP_849398.1 and NP_193428.2), RPP1 (ID: F4J339.1), and Roq1 (ID: ATD14363.1) at the predicted C-JID domain region.

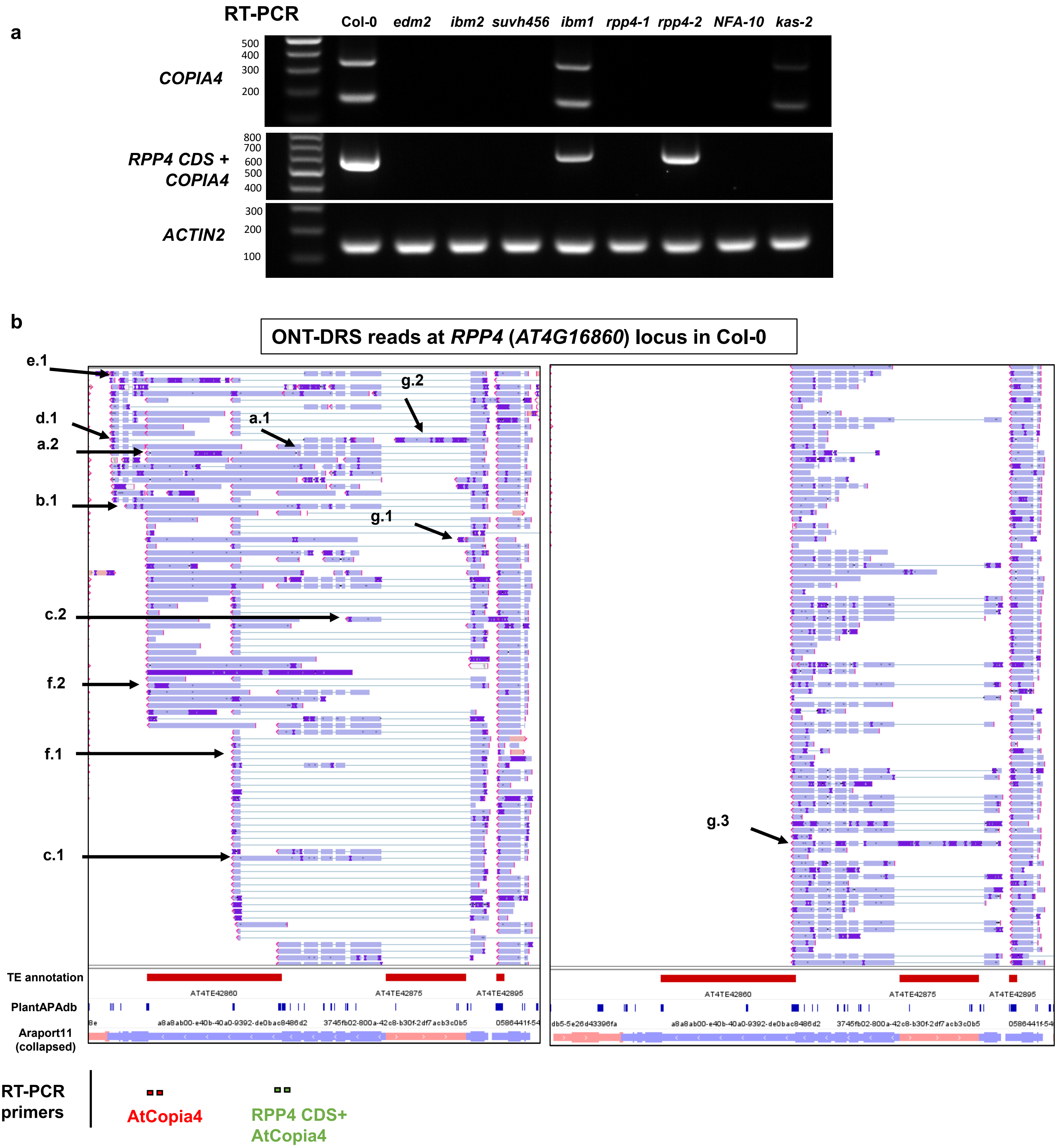

Supplementary Fig. 23. Detection of ATE-G isoforms at the *RPP4* locus. **a)** Agarose gel electrophoresis of RT-PCR amplicons with primers targeting the *ATCOPIA4* or *RPP4-ATCOPIA4* transcribed region. (see Supplementary Fig. 23b for primer positions). *ACT2* (*AT3G18780*) was used as a positive control. **b)** Genome browser tracks showing the alignment of ONT-DRS reads at the *RPP4* locus. Arrows indicate several transcripts classified in the groups A–G shown in Supplementary Fig. 21, which were predicted to encode different protein isoforms. The positions of primers used in Supplementary Fig. 23a are illustrated at the bottom. Source data are provided as a Source Data file.

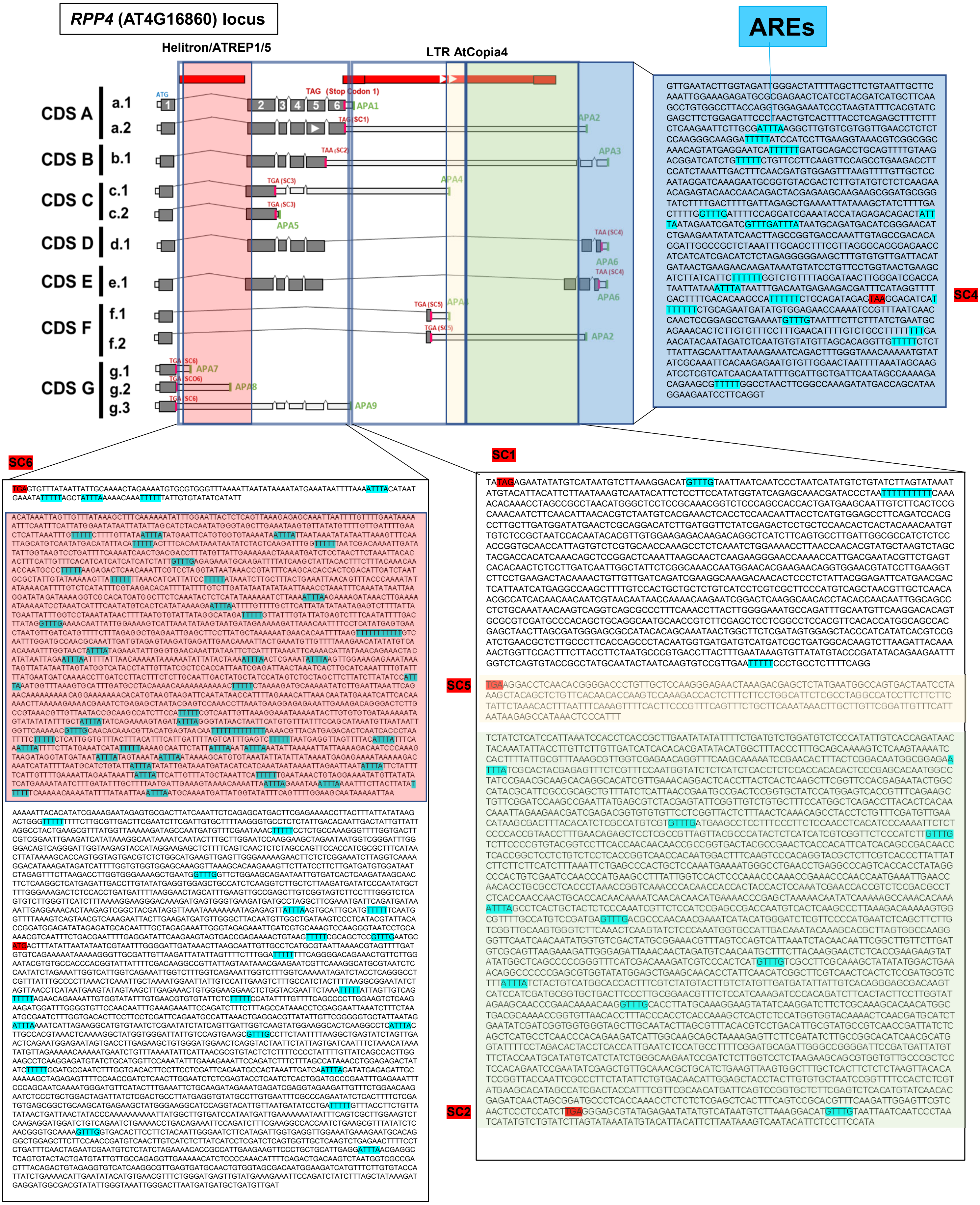

Supplementary Fig. 24. The 3′-UTR of *RPP4* transcripts are predicted to contain AU-rich element (indicated as ARE) motifs. Prediction of potential AU-rich element motifs in the *RPP4* transcripts. SC, predicted stop codons (Supplementary Fig. 21a).

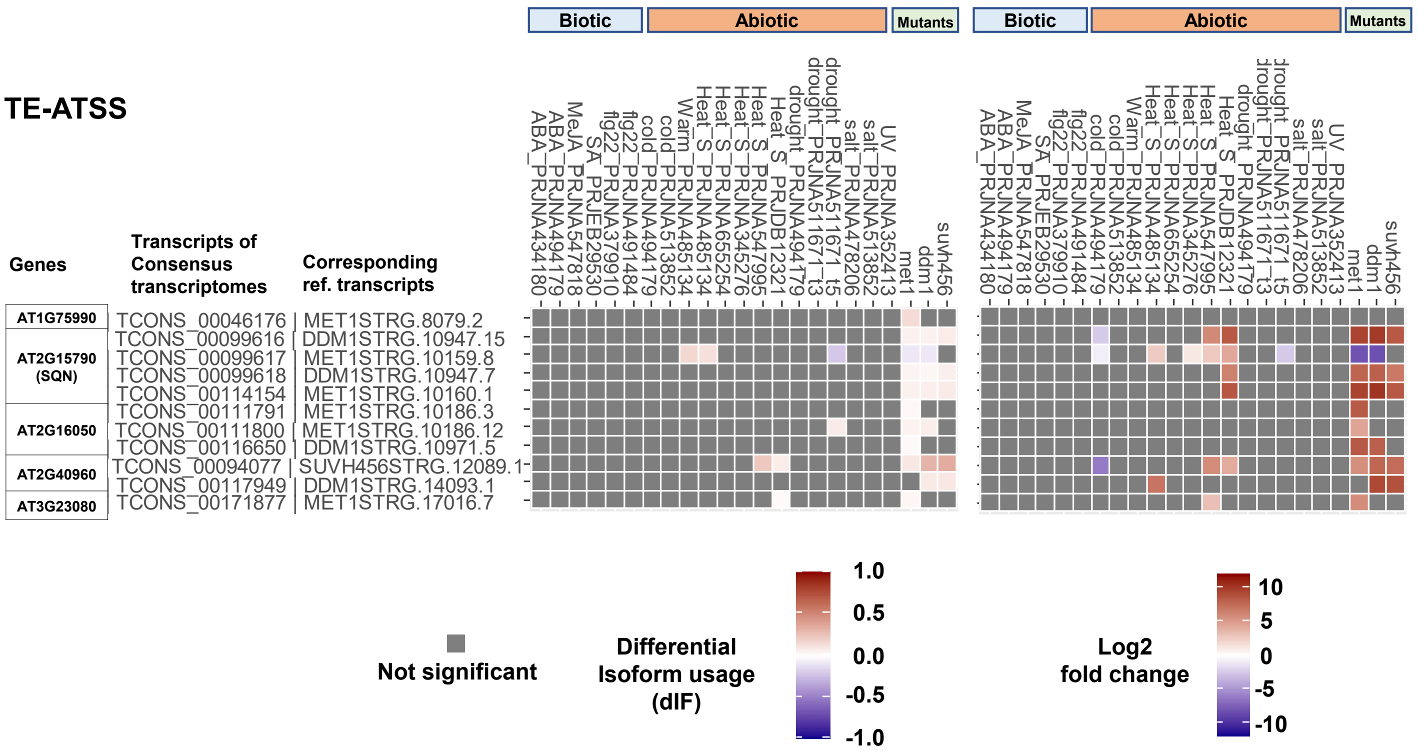

Supplementary Fig. 25. Regulation of Epi-ATE-G isoform candidates with TE-ATSS under various stress conditions. Heatmaps show the statistically significant differential isoform usage (dIF; left) and differential expression fold-change (right) under stress conditions or in the epigenetic mutants. Transcriptome data with stress treatment studies are from the public RNA-seq data. Source data are provided as a Source Data file.

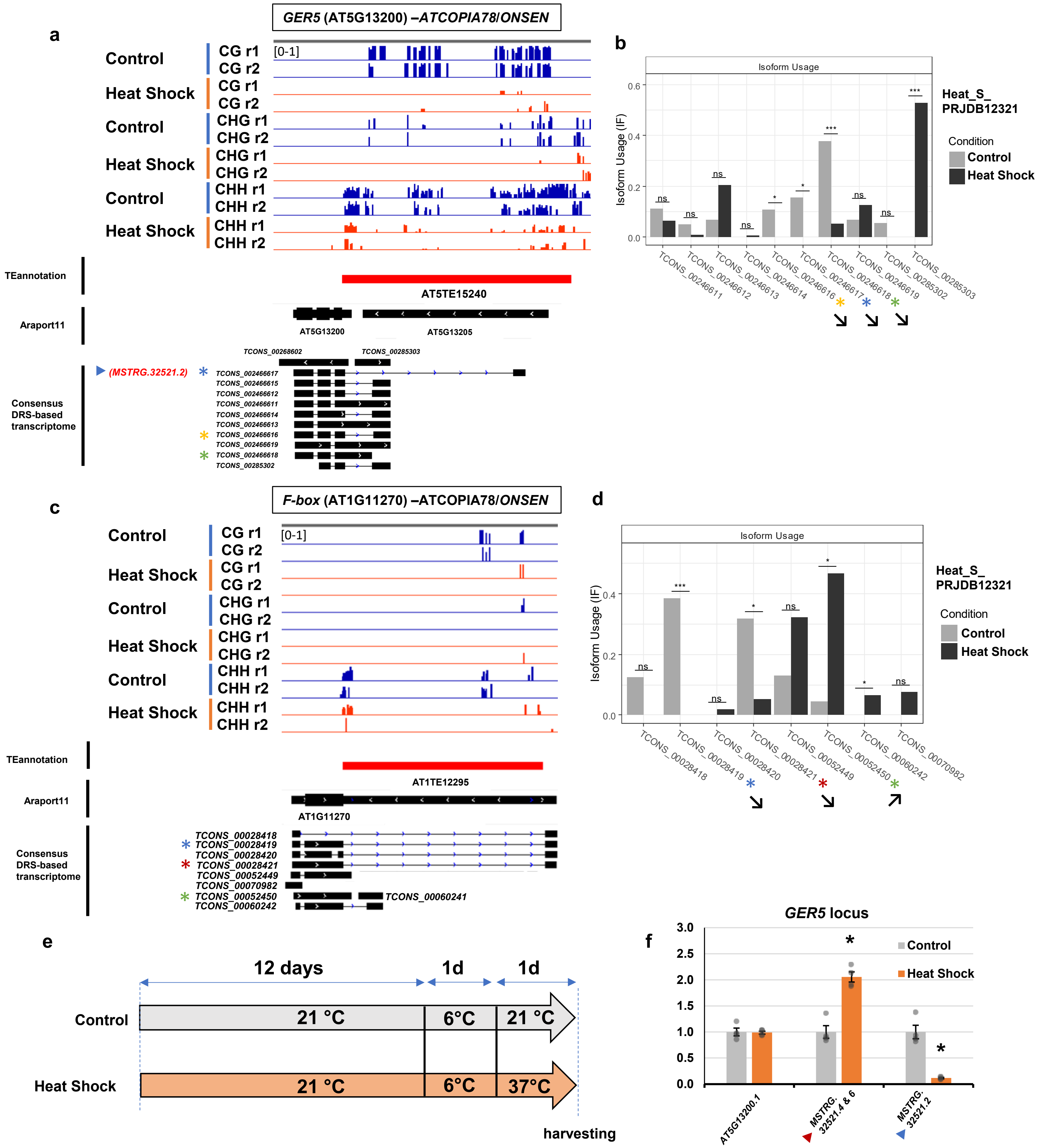

Supplementary Fig. 26. Isoform switching and epigenome changes in *ONSEN*-containing genes under a heat shock treatment. **a)** Changes in CG, CHG and CHH methylation under control and heat shock conditions at *GER5* (*AT5G13200*) locus. Gene annotation in Araport11 and transcripts in the consensus DRS-based transcriptome are displayed. **b)** Changes in isoform usage of ATE-G isoforms associated with the *GER5* locus in the public RNA-seq dataset (PRJDB12321) under a heat shock condition. *, $q < 0.05$; ***, $q < 0.001$ (FDR corrected p-values) obtained by IsoformSwitchAnalyseR. ATE-G isoform with blue asterisk corresponding to the transcript *MSTRG.32521.2,* also marked with a blue arrow in f), showed a significantly lower usage. **c)** Changes in CG, CHG and CHH methylation under control and heat shock conditions at *F-box* gene locus (*AT1G11270*) as in a). **d)** Changes in isoform usage of ATE-G isoforms associated with the *F-box* locus were detected in the public RNA-seq dataset (PRJDB12321) under heat shock condition. *, $q < 0.05$; ***, $q < 0.001$ (FDR corrected p-values) obtained by IsoformSwitchAnalyseR. **e)** Experimental scheme of heat shock treatment applied in this study for qPCR analysis. **f)** Experimental validation of isoform switching under heat shock condition detected with PRJDB12321, PRJNA547995 and PRJNA485134 datasets, displayed in Fig. 7. Relative expression of the *GER5* transcripts (*AT5G13200.1*, *MSTRG.32521.4, .6*, and *MSTRG.32521.2;* see Figure 5a) are shown. Bars represent means of four biological replicates ± SEM and *, $p < 0.05$ by t-test. Source data are provided as a Source Data file.

Supplementary Table 1. Number of corrected "passed reads" and aligned reads (Chr1 to Chr5).

Genotypes	Total corrected reads	Total corrected mapped reads
Col-0 (wt) concatenated with ONT DRS reads from Parker et al 2020	8,336,887	7,547,469
met1	4,134,823	3,974,357
ddm1	5,236,774	5,145,090
suvh456	3,378,954	3,250,179
ibm1	4,136,991	4,030,594
ibm2	3,484,091	3,436,936
edm2	3,727,236	3,678,813

Supplementary Table 2. Number of genes and transcripts in DRS-AtRTD3 and DRS-Araport11 compared to original AtRTD3 and Araport11 (Chr1 to Chr5).

Annotation	Number of genes	Number of transcripts
AtRTD3	40,570	168,949
DRS-AtRTD3	39,998	199,489
Araport11	27,445	48,149
DRS-Araport11	30,552	94,765

Supplementary Table 3. A systematic comparison of DRS-AtRTD3 (199,489 transcripts) against the reference AtRTD3 (Chr1 to Chr5; 10,635 out of 168,949 transcripts were removed as duplicates by the tool criteria, and remaining 158,314 transcripts were analyzed). Only multi-exon transcripts and loci were examined. The sensitivity and precision at base, exon, intron, transcript, and locus levels are shown. Methods and metrics were previously defined[2].

	Sensitivity (%)	Precision (%)
Base level	100.0	91.0
Exon level	92.4	89.1
Intron level	100.0	98.0
Intron chain level	98.8	77.6
Transcript level	97.7	77.5
Locus level	98.0	91.2

Supplementary Table 4. Number and percentage of genes and TEs associated to TE-G transcript and ATE-G isoforms found by ParasiTE in AtRTD3, DRS-AtRTD3, Araport11 and DRS-Araport11.

Gene annotation	AtRTD3	DRS-AtRTD3	Araport11	DRS-Araport11
TE annotation	TAIR10 TE annotation (length ≥200 bp) (18,881)			
Gene associated to TE-G transcripts	2,584 (6.4%)	3,030 (7.6%)	2,358 (8.5%)	2,931 (9.6%)
Gene associated to ATE-G isoforms	1,261 (3.1%)	1,171 (2.9%)	705 (2.5%)	1,082 (3.5%)
TEs associated to TE-G transcripts	2,734 (14.5%)	3,194 (16.9%)	2,511 (13.3%)	2,989 (15.8%)
TEs associated to ATE-G isoforms	1,324 (7.0%)	1,296 (6.9%)	753 (4.0%)	1,171 (6.2%)

Supplementary Table 5. List and detailed of published RNAseq data (paired end) used to investigate the effect of stress conditions on Epi-ATE-G isoforms expression and isoform switching events.

Stress conditions	SRA project	Experiments
ABA (50 µM) 6h	PRJNA434180	Twelve-day-old seedlings were treated with DMSO control or 50 µM ABA for 6 h.
ABA (100 µM) 4h	PRJNA494179	2-week-old seedlings were transferred to 1/2 MS liquid medium with 100 µM ABA for 4h.
MeJA (200 µM) 4h	PRJNA547818	Three-week-old seedlings were grown on MS medium and were treated with mock or 200 µM MeJA for 4 h.
SA (0.5 mM) 24h	PRJEB29530	5-weeks old plants were then sprayed with water or 0.5 mM SA, and harvested after 24 hours.
Flg22 (1 µM) 1h	PRJNA491484	14 days-old seedlings were grown under short-day conditions (8h light/ 16 h dark) at 22°C and treated with 1µM of flg22 for 1h.
Flg22 (1 µM) 0.5h	PRJNA379910	2-week-old seedlings were treated with deionized water (mock) or with a final concentration of 1 µM flg22 for 30 min.
Salt (NaCl)	PRJNA478206	2-week-old seedlings were watered with Hoagland's solution with added 50 mM NaCl. Three days later, they were watered again with Hoagland's with added 100 mM NaCl. The flats were then watered with 150 mM NaCl Hoagland's solution every three days for a total of four additional treatments. Control flats were watered at the same time as salt-treated plants, with solely Hoagland's solution. After treatments, rosette leaves were collected.
Salt NaCl (150 mM) 24h	PRJNA513852	2-week-old seedlings were transferred to Petri dishes containing half MS medium with 1% (w/v) sucrose supplemented with 150 mM NaCl for 24 h.
Warm (28/23 °C) 7d	PRJNA485134	Plants at 23 days after sowing were subjected to 28/23 °C (day/night) for 7 days as the prolonged warming treatment. Leaves were sampled from plants at 30 days after sowing (at the rosette growth stage).
Heat shock (38 °C) 6h	PRJNA485134	Plants were exposed to 38 °C for 6 h during the day portion of the photoperiod as the heat shock treatment. Leaves were sampled from plants at 30 days after sowing (at the rosette growth stage).
Heat shock (37°C) 1h	PRJNA345276	7-day-old seedlings under the normal condition at 22°C (control condition) after an HS treatment at 37°C for 1 h.
Heat shock (37°C) 3x12h	PRJNA547995	7-day-old seedlings were placed in an incubator set at alternating temperatures of 37 and 22 °C, 12 h each, for 3 days with a 12 h photoperiod. Control were at 22 °C.
Heat shock (42 °C) 5h	PRJNA655254	Plants grown in soil under 16 h light/8 h dark conditions for 3 weeks were used for heat treatment at 42 °C for 5 h.
Heat shock (37°C) 24h	PRJDB12321	7-day-old germinated plants sown on MS plates at 21◦C were transferred to a 37◦C incubator. For expression analysis after heat treatment, plants exposed to 37◦C for 24h were sampled immediately following heat treatment and RNA was extracted.
Cold (4 °C) 20 days	PRJNA494179	2-week-old seedlings were transferred to 4 °C and cultured under short-days conditions. Harvested after 20 days.
Cold (4 °C) 1day	PRJNA513852	Seedlings grown under long days at 20°C and transferred to 4°C for 24 h.
Drought 8h	PRJNA494179	2-week-old seedlings were removed from the agar and desiccated in dishes. Harvested after 8 hours.
Drought ADT3 (30–35% moisture)	PRJNA511671	Gradient drought treatments were administered 21 days after planting plants. The soil moistures for Arabidopsis ADT1 plants were 50–55%, 40–45% for ADT2, 30–35% for ADT3, 20–25% for ADT4, 10–15% for ADT5.
Drought ADT5 (10–15% moisture)	PRJNA511671	
UV-B 24h	PRJNA352413	15-day-old seedlings were exposed to UV-B light (0,210 mW/cm2) during 24 h and then recovered for 72 h under controlled conditions (16 h light, 100 µmoles m−2 s−1, 22 ± 2ºC). As control, seedlings covered with a cellulose acetate polyester filter were used.

Supplementary Table 6. RT-qPCR Primers used in this study.

Name	Primer sequence 5` → 3`	Primer efficiency
BEST1_cds_P0_F	CTTTCCCTGTTGCTCTTAAGTGTC	104.3
BEST1_cds_ P0_R	CAAGTTCCGGAGATCTCTTGCT	
BEST1_ P1_F	GGGAGCTAAGAACATGTGATGG	
BEST1_ P1_R	CACTTAAACACTTTCTCCATTACCTC	
AT4G16900_ cds_ P0_F	CGTACCTCACGTCTATAGCATGAAG	
AT4G16900_ cds_ P0_R	ACTTCCCTCCTATCTTTATATCCTTACC	
AT4G16900_ P1_F	CGTTACAGATAACCTGCCCTACAG	
AT4G16900_ P1_R	GAGTATACTCGTCATCGTTTCTGG	
AT4G16900_ P2_F	CTGGTAAGGACATAGGCTTGAAC	
AT4G16900_ P2_R	CCTTTCATTATCTCTTCCAGAACTG	
GER5_cds_AT5G13200.1&2_F	CTCTGGCACAATCTGAAGACAG	
GER5_cds_AT5G13200.1&2_R	GCTCTGTTCCGAAAATCTGTCTG	
GER5_cds_AT5G13200.1_F	GGAACTGTCTATCTTTCGAATGC	104.5
GER5_cds_ AT5G13200.1_R	GGTACAACCACCCTGTAGTAGCTC	
GER5_ MSTRG.32521.4&.6_F	CATCTGCTGACCAGTGTCTCC	
GER5_ MSTRG.32521.4&.6_R	ACATTGACACACGCTATCACTCTA	
GER5_ MSTRG.32521.2_F	CATCTGCTGACCAGTGTCTCC	
GER5_MSTRG.32521.2_R	CGTGGGAAAGGACACCTCTA	
ACT2_F	GATGGAAACCTCAAAGACCAG	102.0
ACT2_R	CACAAACGAGGGCTGGAAC	
GAPDH_(AT1G13440)_F	CGTTGACCTTATCGTTCACATG	102.0
GAPDH_(AT1G13440)_R	CAGAACAGTACGAACTCAACCAC	
RPP4_cds_ P0_F	CCACGGTATTATTTTCGACAAG	100.4
RPP4_cds_ P0_ R	GGTAGATCTATTTTTGACCAAAGACC	
RPP4_ P1_F	CAAACATGTCCTTTAAGACATTATGAC	
RPP4_ P1_R	CGACGTATATTGGGTAAATTGG	
RPP4_ P2_F	CTTGAAGCATGATCGTAGGATG	
RPP4_ P2_R	CCATAGTTGAATACTTGGTAGATTGG	
RPP4_ P3_F	GATGTCATCTGGATGCCTTCC	
RPP4_ P3_R	GTGAATTTCGCCCAGAATATCTC	
RPP4_ P4_F	GAGGTCCTTCACCCGTTCC	
RPP4_ P4_R	CGAGGACAAACCAGAGGATC	
RPP4_ P5_F	CACGCACATTTTCTAGTTTTGC	
RPP4_ P5_R	GATCTTCGGAACGGGTATG	
AIG2-like_(AT3G28950)_cds_P0_F	GGTCTTAAGCCCAAAAGAGGG	
AIG2-like_(AT3G28950)_cds_P0_R	GCAATAGTTGCTTCGGAGTGG	
AIG2-like_(AT3G28950)_P1_F	GGTCTTAAGCCCAAAAGAGGG	
AIG2-like_(AT3G28950)_P1_R	GCAATAGTTGCTTCGGAGTGG	

Supplementary Table 7. RT-PCR Primers used in this study.

Name	Primer sequence 5` → 3`
ACT2_RT-PCR_F	AACTCTCCCGCTATGTATGTCGCCATCCAA
ACT2_RT-PCR_R	AGCAAGGTCAAGACGGAGGATGGCATGAGG
GER5_RT-PCR_F	CATCTGCTGACCAGTGTCTCC
GER5_RT-PCR_R	CCAAAGAAGAGGTGGTGGTC
BEST1_RT-PCR_P2_F	GGGAGCTAA GAACATGTGATGG
BEST1_RT-PCR_P2_R	GTTAGACTAATCATCTCACTAGC
ATCOPIA4_RT-PCR_F	TTGACGCCCAACAACGAAAT
ATCOPIA4_RT-PCR_R	GTTTCCGCATAGTCGACACC
RPP4_CDS_ATCOPIA4_F	CGATAGAACCATCAAGATGTCCT
RPP4_CDS_ATCOPIA4_R	GAGTGATGCAACTGTGGTAGC

Supplementary Note 1. Detection of TE-gene transcripts by the ParasiTE pipeline.

Input data for the ParasiTE pipeline:

The following four input data were used for ParasiTE analysis in this study:

1) Transcriptome annotation of *A. thaliana* Araport11 (https://phytozome-next.jgi.doe.gov/info/Athaliana_Araport11, "Athaliana_447_Araport11.gene_exons.gff3")[3] or AtRTD3[4] or DRS-Araport11 or DRS-AtRTD3 or mutant-DRS transcriptomes obtained with Stringtie2[5].

2) Gene model annotation of *A. thaliana* Araport11 (https://phytozome-next.jgi.doe.gov/info/Athaliana_Araport11, "Athaliana_447_Araport11.gene.gff3")[3] or AtRTD3 (https://ics.hutton.ac.uk/atRTD/RTD3/)[4]. Gene and transcript feature of AtRTD3 gtf were retrieved using gffread (v0.12.2; parameters: --keep-genes)[6].

3) TE annotation of *A. thaliana* of TAIR10 (retrieved from the URGI laboratory browser; https://urgi.versailles.inra.fr/gb2/gbrowse/tairv10_pub_TEs/)[7]. Only TEs with a length ≥200 bp were analyzed in this study.

4) A gene-like TE annotation retrieved from TAIR (ftp://ftp.arabidopsis.org/home/tair/Genes/TAIR10_genome_release/TAIR10_gff3/TAIR10_GFF3_genes_transposons.gff) and published gene-like TE annotation data by Panda et al.[8] were concatenated.

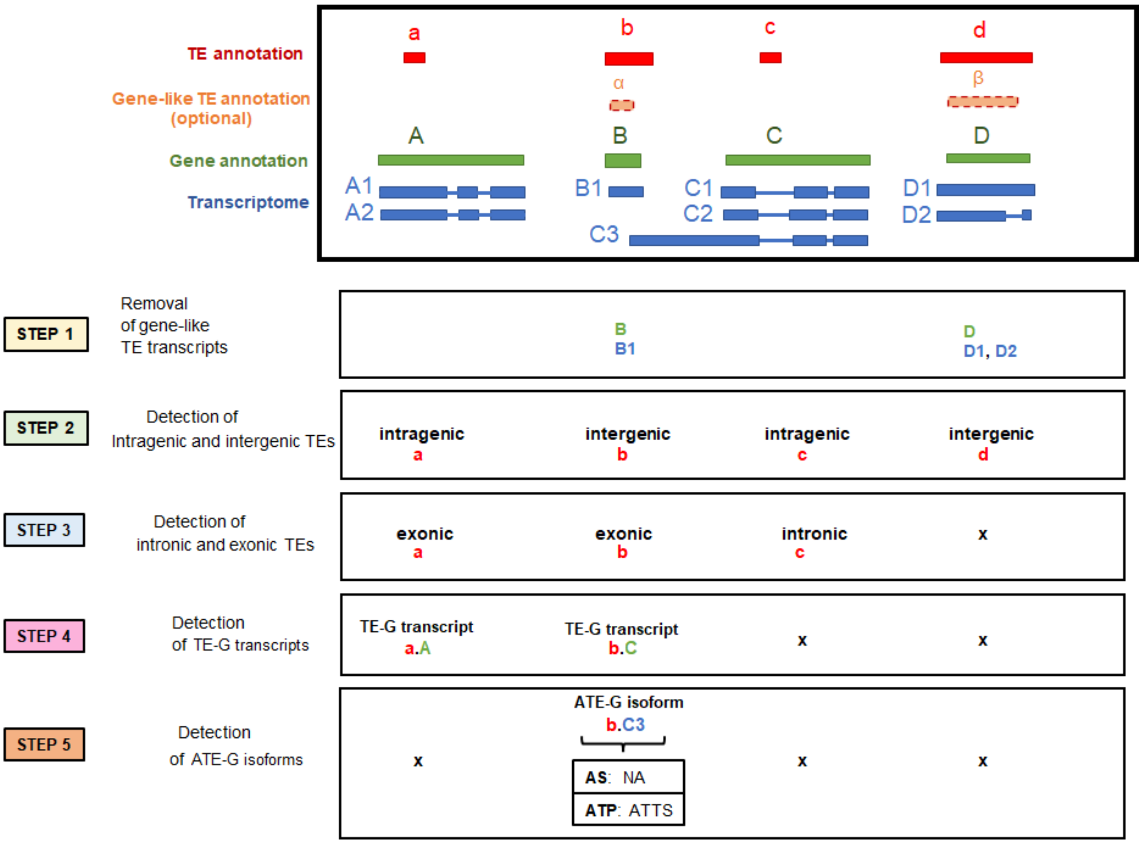

Supplementary Fig. 27. Main steps of the ParasiTE pipeline.

Step1: Removal of gene-like TEs

ParasiTE first detects gene models of active TEs called "gene-like TEs"[8]. Next, it removes the annotation of gene-like TEs from the gene model annotation and the associated transcripts in the transcriptome annotation (Supplementary Fig. 27). Indeed, gene-like TEs are the major cause of false positive events in the detection of TE-gene (TE-G) transcripts or alternative TE-gene (ATE-G) isoforms in downstream analysis.

For this task, ParasiTE uses bedtools' intersect function[9] to compare the gene model annotation with the provided TE annotation. ParasiTE detects gene annotations overlapped by TEs by at least 80% in length of the gene annotation. Those gene annotations are considered to be gene-like TEs. ParasiTE generates a list of these gene models and removes them from the original gene model annotation. Next, ParasiTE uses the created list of gene models, defined as gene-like TEs, to remove transcripts (and associated exons) in the transcriptome dataset that overlap their annotation by at least 80% in length.

Optionally, a gene-like TE annotation can be provided. In this case, ParasiTE applies the second round of filtering and identifies leftover transcripts that overlap the provided gene-like TE annotation by at least 80% in length and removes the transcript (and associated exon) annotations from the transcriptome dataset. This step may help to detect gene-like TEs that have been imprecisely identified in the TE annotation (for example, because of fragmented TE annotation). This step improves the mis-annotation of gene-like TEs as TE-G transcripts (Supplementary Fig. 40).

Step 2: Detection of transcripts containing intragenic and intergenic TEs

In the next step, ParasiTE classifies TEs according to their location based on the gene annotation (Supplementary Fig. 27). This step employs the bedtools intersect function[9]. TEs that overlap a gene by at least 80% in length are regarded as "intragenic TE", and the remaining TEs as "intergenic TEs". Then, ParasiTE looks for intergenic TEs close to genes potentially involved in TE-G transcript formation. Intergenic TEs within 2 kb of an annotated gene are extracted by bedtools' closest function[9] for further processing. Three rounds of extraction are applied at each side of the TE.

Step 3 and 4: Annotation of transcripts containing exonic and intronic TEs

Next, ParasiTE compares the intragenic and selected intergenic TEs to the transcriptome dataset with the bedtools intersect function[9] to annotate TEs that are fully exonic, partially exonic, or intronic. Two methods are applied to identify "exonic TEs", with method 1 identifying TEs overlapping exons by at most 80% in length, while method 2 detects TEs that overlap more than 80% the length of an exon. Next, method 3 detects leftover TEs that overlap exons by at least 1% in length and are classified as "partially exonic TEs" (Supplementary Fig. 28). Intragenic TEs not classified by either of these methods and which do not overlap by 1% or more an exon are considered as "intronic TEs".

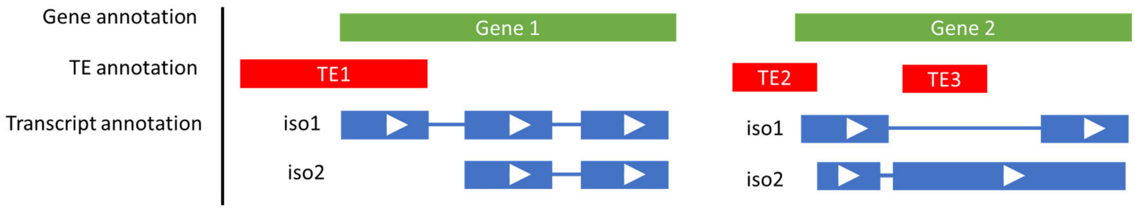

Supplementary Fig. 28: Schematic illustration of different types of exonic TEs and methods used to find them. TE3 is detected by method 1, TE1 by method 2, and TE2 by method 3.

Step 4 and 5: Identification of TE-G transcripts and ATE-G isoforms

At this step, intragenic and intergenic TEs defined as “exonic TEs” or “partially exonic TEs" are used as TE-G transcript candidates. Intergenic TEs may be identified as TE-G transcript candidates if they were not properly annotated as part of the gene model annotation.

ParasiTE generates a list of candidate TEs and the associated transcripts and exons with which they form TE-G transcripts. More precisely, ParasiTE lists every pair of TE and overlapping exon as TE-G transcript candidates.

For example: "AT1G03410.1_845951_847683" (Exon id) & AT1TE02770 (TE id).

Next, ParasiTE takes advantage of the tool CATANA[10] to identify ATE-G isoforms among TE-G transcript candidates in the transcriptome dataset. Using the transcriptome annotation (.gtf or .gff) as an input, CATANA detects and classifies the occurrence of alternative splicing (AS) and alternative transcription product (ATP) events for each exon, transcript, and gene. CATANA finds more AS and ATP events than the tools MISO[11] or ASprofile[12] and has the advantage of detecting both AS and ATP events[10].

ParasiTE uses this information to retrieve exon-TE pairs involved in ATE-G isoforms. ParasiTE retrieves AS and ATP predictions from CATANA for exon-TE pair events (Supplementary Fig. 27, step 5). This information is first given as raw output data and combined by ParasiTE to summarize the events appearing for every TE-gene pair. Although CATANA[10] can identify single and multiple skipping exons, this information is combined as “exon skipping” (ES) events in ParasiTE. Moreover, this first version of ParasiTE does not consider mutually exclusive exon (MXE) events found by CATANA[10].

ParasiTE eventually identifies alternative TE-related splicing sites (TE-AS) composed of alternative TE-related 5’/3’ splice sites (TE-A5SS/TE-A3SS), TE-related

exon skipping (TE-ES), and TE-related intron retention (TE-IR). Moreover, it can identify TE-related ATPs composed of TE-related alternative transcriptional start/termination sites (TE-ATSS/TE-ATTS), and among those, it predicts TE-related alternative first/last exons (TE-AFE/TE-ALE; Supplementary Fig. 29).

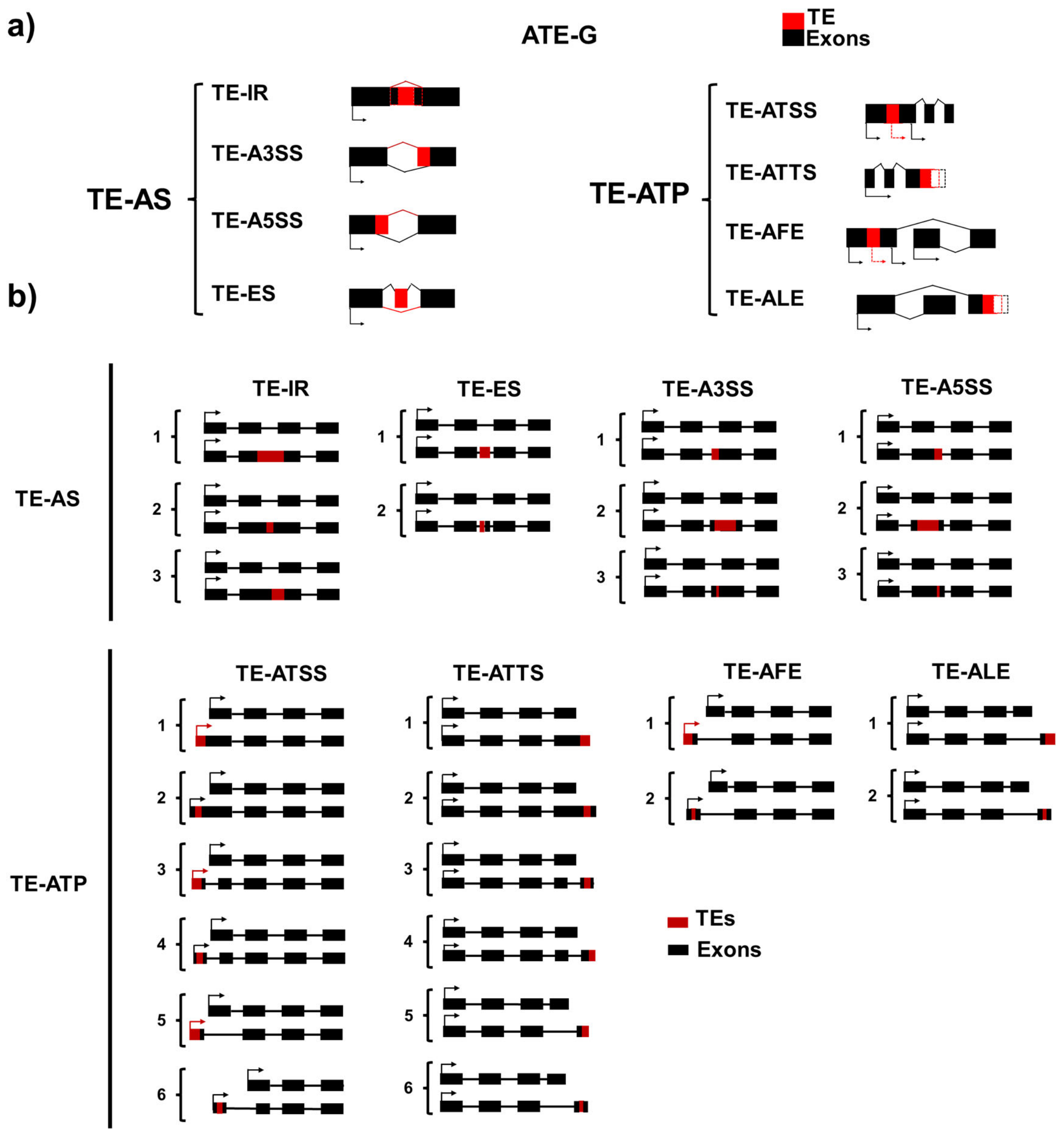

Supplementary Fig. 29: a) Schematic illustration of TE-AS and TE-ATP events identified by ParasiTE. b) Illustration of ATE-G isoforms considered as positive predictions by ParasiTE using the CATANA classification[10]. Several cases of positive predictions are displayed for each type of TE-AS and TE-ATP event.

Filtering used during step 5

Step 5 introduces potential false positive events because CATANA[10] may give unprecise predictions of AS or ATP events at the exonic level. Indeed, we noticed that CATANA may predict false positive AS or ATP events for an exon if a neighboring exon features these events. Moreover, an exon overlapping with a TE may be regarded as an AS or ATP event even if it is not directly related to the TE (Supplementary Fig. 30). This may introduce false positives during the prediction of ATE-G isoforms processed by ParasiTE.

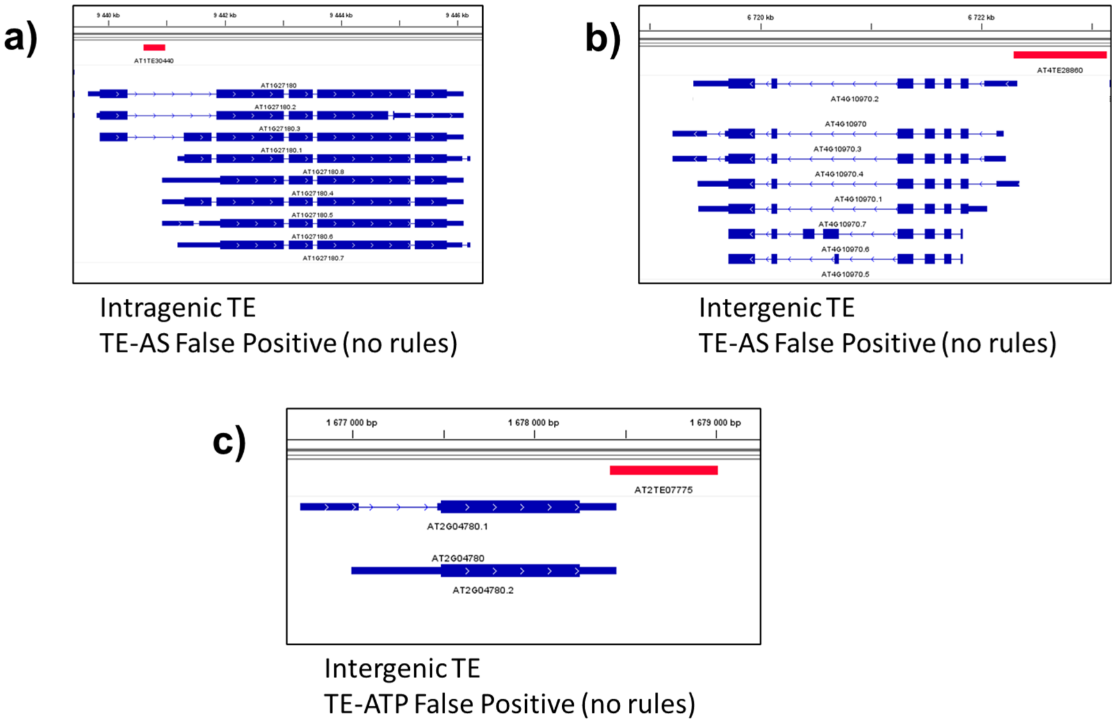

Supplementary Fig. 30: Examples of false positive predictions of TE-AS or TE-ATP events without the application of filtering rules. a) and b) False positive TE-AS prediction by ParasiTE (without filtering rules). CATANA found an IR event for the nearby exon that was not related to the TE sequence. c) False positive TE-ATP prediction (TE-ATSS) by ParasiTE (without filtering rules). CATANA found an ATSS event for the overlapping exons, because of the neighboring exon features this event, but this ATSS event was not related to the TE sequence.

Therefore, several filtering rules were further implemented in ParasiTE to minimize false positive predictions of TE-AS or TE-ATP events. These rules use splicing site information

of transcripts retrieved using the "extract_splice_sites.py" script from Hisat2[13]. ParasiTE also uses information of the location of the TE (intragenic or intergenic) to check for overlapping between the TE and edge of the associated exon and the frequency with which the TE overlaps transcripts of the same associated gene (noted “F”). Rules are illustrated below (Supplementary Fig. 31-37).

Rule 1: A TE-AS event(s) is/are not predicted for a TE that does not overlap any splicing site(s), and that overlaps all isoforms of the gene (i.e., frequency of overlapping with isoforms of the associated gene is 100%, F = 1; Supplementary Fig. 31).

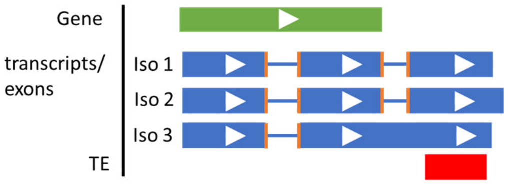

Supplementary Fig. 31: Example of rule 1. Orange lines represent splicing sites.

Rule 2: A TE-ATP event(s) is/are not predicted for an intragenic TE that overlaps with all isoforms of the gene (F = 1), is located at the first or last exon of the transcript, and does not overlap with the edge of the associated exon (Supplementary Fig. 32).

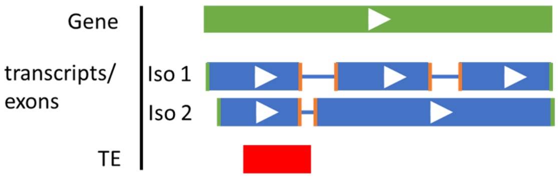

Supplementary Fig. 32: Example of rule 2. Orange lines represent spicing sites and green lines represent the edge of the exon.

Rule 3: A TE-ATP event(s) is/are not predicted for an intragenic TE that has a TE-AS prediction, does not overlap with a splicing site, and does not overlap with the edge of the overlapping exon(s) (Supplementary Fig. 33).

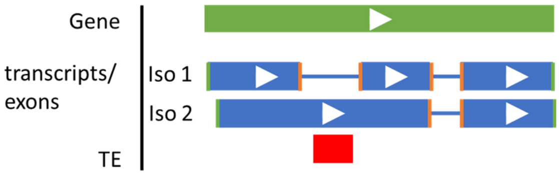

Supplementary Fig. 33: Example of rule 3. Orange lines represent spicing sites, and

green lines represent the edge of the exon.

Rule 4: A TE-AS event(s) is/are not predicted for an intragenic TE that has a TE-ATP prediction, does not overlap with any splicing site, and overlaps with a single-exon transcript or the first or last exon of the associated transcript (Supplementary Fig. 34)

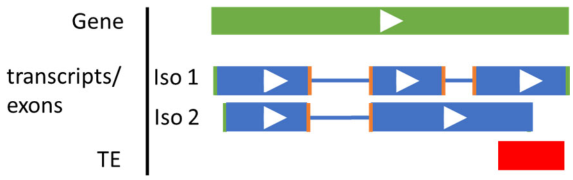

Supplementary Fig. 34: Example of rule 4. Orange lines represent spicing sites, and green lines represent the edge of the exon.

Rule 5: A TE-AS event(s) is/are not predicted for an intergenic TE which does not overlap with any splicing site and overlaps with the first or last exon of the associated transcript (Supplementary Fig. 35).

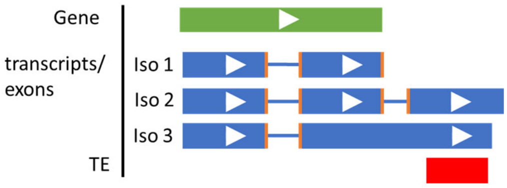

Supplementary Fig. 35: Example of rule 5. Orange lines represent spicing sites, and green lines represent the edge of the exon.

Rule c1: TE-A5SS and/or TE-A3SS is/are not predicted for a TE that is not overlapping with the right side of the associated exon (Supplementary Fig. 36).

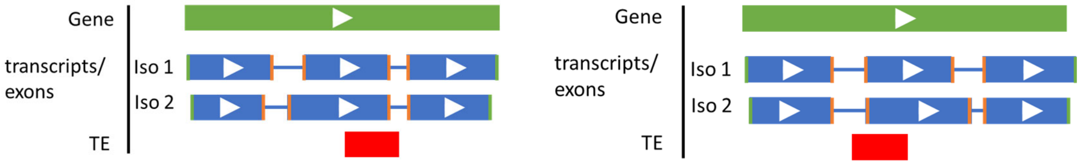

Supplementary Fig. 36: Example of rule c1. Orange lines represent spicing sites, and green lines represent the edge of the exon. Left: a false positive TE-A3SS event; right: a false positive TE-A5SS event.

Rule c2: A TE-ATP event(s) is/are not predicted for a TE composed of one, two, or three exons and is not overlapping with the edge of the associated transcript (Supplementary Fig. 37). This rule removes false positives displayed in Supplementary Fig. 30c.

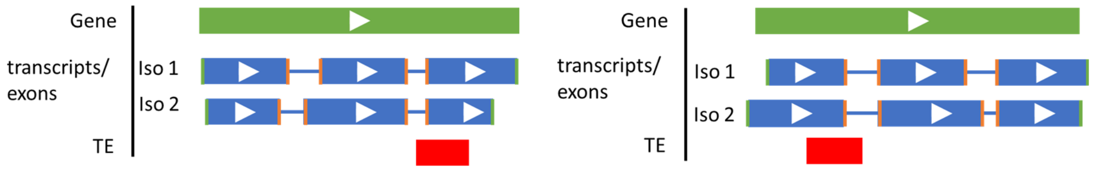

Supplementary Fig. 37: Example of rule c2. Orange lines represent spicing sites, and green lines represent the edge of the exon.

<u>Output of ParasiTE</u>

ParasiTE generates output files that list TE-G transcript and ATE-G isoform candidates at the exonic level (with transcript level information) and gene level.

Evaluation of the ParasiTE pipeline using *A. thaliana* gene annotation

We evaluated the accuracy of the ParasiTE pipeline with the Araport11 transcriptome dataset, using gene model and TE annotations, transcriptomic data, and gene-like TE annotation as described above. We first ran ParasiTE without applying any filtering rules (no rules) and manually checked the prediction of TE-AS and/or TE-ATP events for each TE-gene pair. We labeled as true positive or false positive the TE-AS or TE-ATP predictions of each TE-gene pair. Moreover, because TE-Gene pair associated to ATE-G isoforms may be involved only in TE-AS but not in TE-ATP (or vice-versa), we also checked the "NA" predictions for TE-AS and TE-ATP (example of TE-AS: "NA" and TE-ATP: "ATTS" in Supplementary Fig. 27). A correct "NA" prediction was labeled as a true negative, but an incorrect "NA" prediction was labeled as a false negative. In this analysis, we only checked if TEs were directly involved in TE-AS or TE-ATP events without checking the accuracy of the type of events (such as TE-IR or TE-A3SS). For ATE-G isoforms associated to intragenic TEs we checked events found in chromosomes 1 to 5 (300 ATE-G isoform events with no rules) of the *Arabidopsis* genome. For ATE-G isoforms associated to intergenic TEs we checked events found in chromosomes 1 and 2 (259 ATE-G isoform events with no rules), but five ATE-G isoform events were manually found associated to potential gene-like TEs and were removed, resulting in 254 ATE-G isoform events (with no rules). Next, we applied the five filtering rules (see above) to the ParasiTE pipeline to minimize false positive predictions (Supplementary Fig. 38). We obtained reasonably good positive predictive values (PPV) for TE-AS and TE-ATP by applying rules 1-5, which achieved a total (intergenic and intragenic TEs) PPV of ~78% for TE-AS and ~96% for TE-ATP events (Supplementary Fig. 38a). Applying rules 1-5 did not affect the NPV of ~100% for TE-AS or TE-ATP events (Supplementary Fig. 38b).

a)

b)

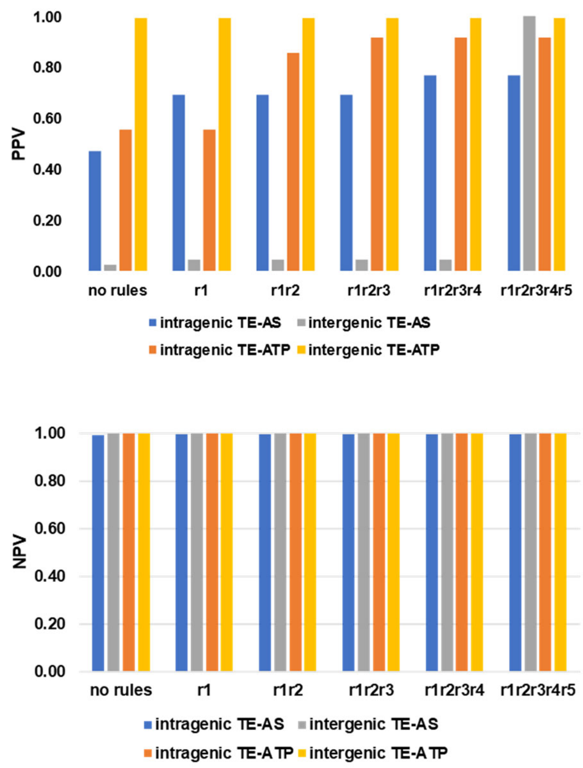

Supplementary Fig. 38: a) Positive predictive values (PPV) and b) Negative predicted values obtained by ParasiTE with the Araport11 annotation. Filtering rules (from no rules to rules 1, 12, 123, 1234, and 12345) improved the detection accuracy of ATE-G isoform events.

Next, we further evaluated the tool's accuracy for predicting ATE-G isoform formation events by distinct molecular mechanisms (Supplementary Fig. 29a). TE-AS events can be further classified into splicing events, including TE-IR, TE-ES, TE-A5SS, and TE-TE-A3SS, while TE-ATP events can be further classified into TE-ATSS, TE-ATTS, TE-AFE, and TE-ALE. For this analysis, predictions for potential gene-like TEs were not removed in order to measure their impact on the tool's accuracy.

After manual inspection of predicted TE-AS events (n = 144 after c1c2) in chromosomes 1 and 2 of the *Arabidopsis* genome, we found that 60% of TE-ES (n = 10 after c1c2), 77% of TE-IR (n = 88 after c1c2), 70% of A5SS (n = 20 after c1c2), and 46% of A3SS (n = 26 after c1c2) events were correctly predicted (Supplementary Fig. 39). In addition, we found that 96% of ATSS (n = 150 after c1c2), 94% of ATTS (n = 148 after c1c2), 93% of AFE (n = 43 after c1c2), and 87% of ALE (n = 15 after c1c2) events were correctly predicted (Supplementary Fig. 39). Overall, the ParasiTE pipeline can reasonably identify and classify ATE-G isoform formation events.

a)

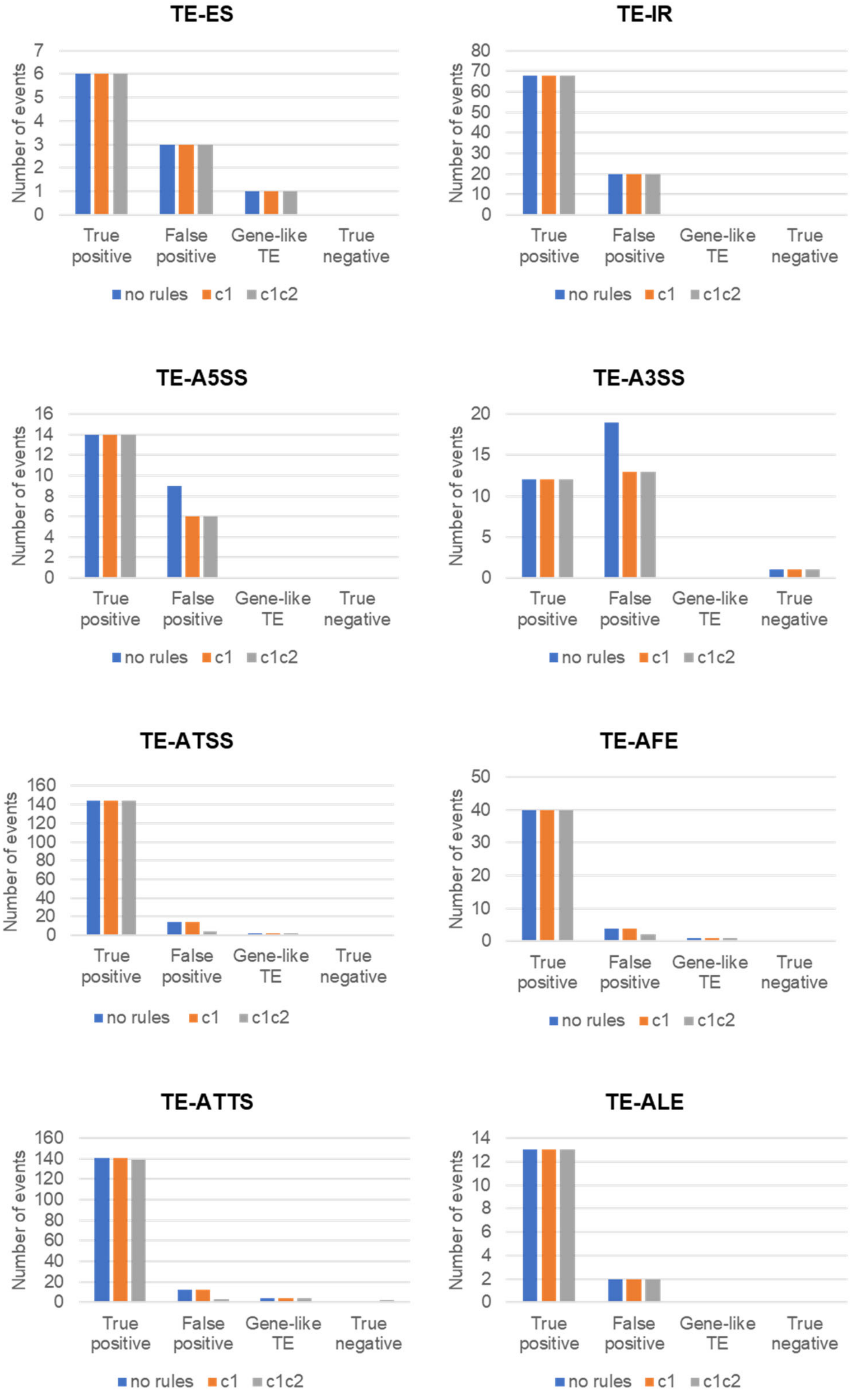

TE-ES
TE-IR
TE-A5SS
TE-A3SS
TE-ATSS
TE-AFE
TE-ATTS
TE-ALE
Number of events
True positive
False positive
Gene-like TE
True negative
no rules
c1
c1c2

b)

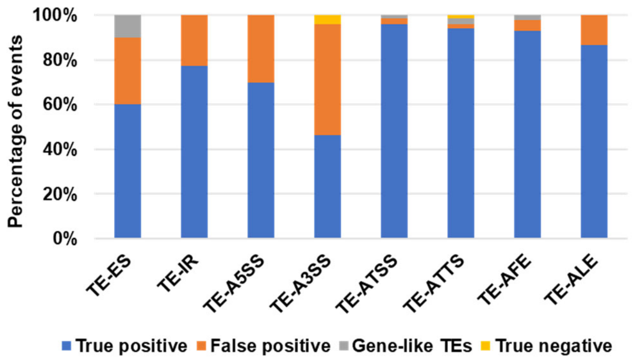

Supplementary Fig. 39: a) Evaluation of prediction efficiency of distinct TE-AS and TE-ATP events obtained by ParasiTE with the Araport11 annotation and either with (rules c1 and c2) or without (no rules) correction rules. b) Evaluation of the efficiency of ParasiTE to predict TEs involved in ATE-G isoform (TE-AS or TE-ATP) events.

We also evaluated the efficiency of the removal of gene-like TEs in step 1 of the ParasiTE pipeline (see Supplementary Fig. 40) using an optional "gene-like TE annotation". A comparison of ParasiTE outputs obtained from the Col-0 DRS and *ddm1*-DRS data (which contains many activated "free" gene-like TE transcripts) showed that the step greatly reduced false positive predictions of TE-G transcripts and improved the prediction of true events (Supplementary Fig. 40). In the *ddm1*-DRS dataset, numerous TE transcripts are included, which increased the number of false positive detections of TE-AS and TE-ATP events. The optional filtering step using a "gene-like TEs annotation" helped to reduce this number (orange bars).

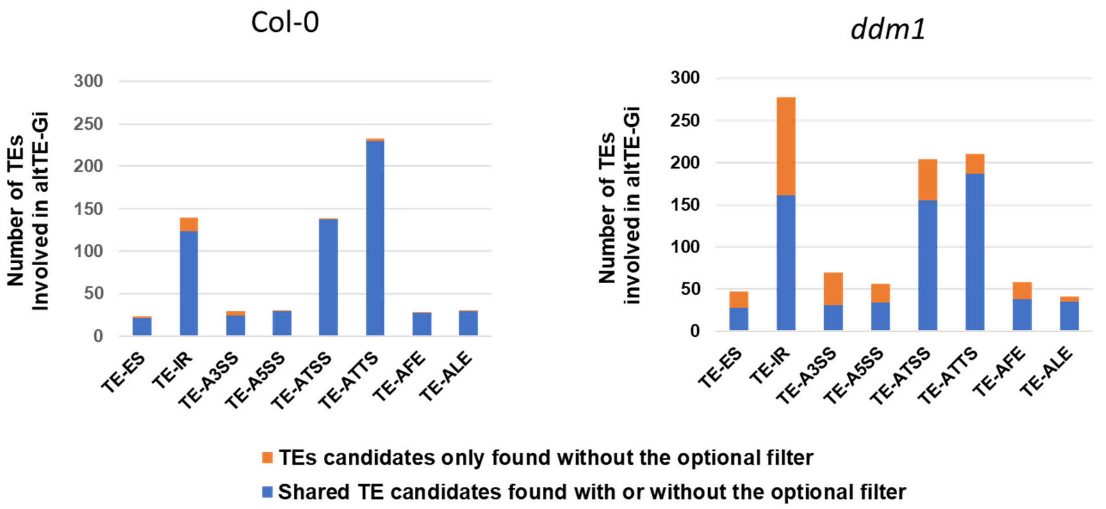

Supplementary Fig. 40: Number of TEs involved in ATE-G isoform events found by ParasiTE using the *de novo* DRS transcriptome of Col-0 and *ddm1* (Stringtie2 "-L mode" and Araport11 as the reference). Blue: number of TEs predicted using the optional filter with a gene-like TE annotation. Orange: additional TEs predicted as being involved in ATE-G isoform events without the filter; these correspond to gene-like TEs and are false positive predictions. Many gene-like TEs were retrieved in *ddm1* because of the lack of DNA methylation, causing TE re-activation.

Finally, ParasiTE can estimate the contribution of TE-G transcripts and ATE-G isoforms to CDS or 5′/3′-UTRs. For each TE-G transcript events, ParasiTE uses bedtools' intersect function to overlap each exonic region of TEs involved in TE-G transcripts to the CDS and 5'/3'-UTRs of genes. Features per gene can be counted, as demonstrated in Supplementary Fig. 41**.**

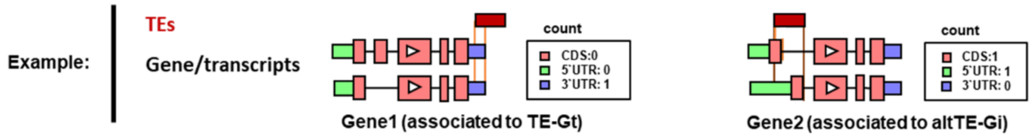

Supplementary Fig. 41: Example of ParasiTE's counting feature.

References:

1. Stroud, H., Greenberg, M. V., Feng, S., Bernatavichute, Y. V. & Jacobsen, S. E. Comprehensive analysis of silencing mutants reveals complex regulation of the Arabidopsis methylome. Cell 152, 352–364 (2013).
2. Pertea, G. & Pertea, M. GFF utilities: GffRead and GffCompare. F1000Research 9, (2020).
3. Cheng, C. et al. Araport11: a complete reannotation of the Arabidopsis thaliana reference genome. The Plant Journal 89, 789–804 (2017).
4. Zhang, R. et al. A high-resolution single-molecule sequencing-based Arabidopsis transcriptome using novel methods of Iso-seq analysis. Genome Biology 23, 149 (2022).
5. Kovaka, S. et al. Transcriptome assembly from long-read RNA-seq alignments with StringTie2. Genome Biol 20, 278 (2019).
6. Pertea, G. & Pertea, M. GFF utilities: GffRead and GffCompare. F1000Research 9, (2020).
7. Buisine, N., Quesneville, H. & Colot, V. Improved detection and annotation of transposable elements in sequenced genomes using multiple reference sequence sets. Genomics 91, 467–475 (2008).
8. Panda, K. & Slotkin, R. K. Long-read cDNA Sequencing Enables a 'Gene-Like' Transcript Annotation of Arabidopsis Transposable Elements. http://biorxiv.org/lookup/doi/10.1101/2020.02.20.956714 (2020) doi:10.1101/2020.02.20.956714.
9. Quinlan, A. R. & Hall, I. M. BEDTools: a flexible suite of utilities for comparing genomic features. Bioinformatics 26, 841–842 (2010).

10. Shiau, C.-K., Huang, J.-H. & Tsai, H.-K. CATANA: a tool for generating comprehensive annotations of alternative transcript events. Bioinformatics 35, 1414–1415 (2019).

11. Katz, Y., Wang, E. T., Airoldi, E. M. & Burge, C. B. Analysis and design of RNA sequencing experiments for identifying isoform regulation. Nature Methods 7, 1009–1015 (2010).

12. Florea, L., Song, L. & Salzberg, S. L. Thousands of exon skipping events differentiate among splicing patterns in sixteen human tissues. F1000Research 2, (2013).

13. Kim, D., Paggi, J. M., Park, C., Bennett, C. & Salzberg, S. L. Graph-based genome alignment and genotyping with HISAT2 and HISAT-genotype. Nature Biotechnology 37, 907–915 (2019).
