# Supplementary material for: Long-read direct RNA sequencing reveals epigenetic regulation of chimeric gene-transposon transcripts in Arabidopsis thaliana
Source: Nat Commun. 2023 Jun 5;14:3248. doi: 10.1038/s41467-023-38954-z (PMC10241880; doi:10.1038/s41467-023-38954-z)
Supplement: Supplementary file 3 — Description of Additional Supplementary Files [file 41467_2023_38954_MOESM3_ESM.pdf]

Description of Additional Supplementary Files

Supplementary Data 1: Properties of the ONT sequencing data.

Supplementary Data 2: Outputs of ParasiTE for DRS-Araport11 and DRS-AtRTD3.

Supplementary Data 3: Enrichment analysis of *A. thaliana* TE superfamilies in ATE-G isoform and three TE families in TE-IR events.

Supplementary Data 4: Change in percentage of DNA methylation in TEs between Col-0 and epigenetics mutants.

Supplementary Data 5: Isoform switch analysis of ATE-G isoform candidates using DRS-AtRTD3 as background.

Supplementary Data 6: Isoform switch analysis of ATE-G isoform candidates using ONT-DRS mutant transcriptomes as background.
