# Supplementary material for: Long-read direct RNA sequencing reveals epigenetic regulation of chimeric gene-transposon transcripts in Arabidopsis thaliana
Source: Nat Commun. 2023 Jun 5;14:3248. doi: 10.1038/s41467-023-38954-z (PMC10241880; doi:10.1038/s41467-023-38954-z)
Supplement: Supplementary file 10 — Reporting Summary [file 41467_2023_38954_MOESM10_ESM.pdf]

nature portfolio

Corresponding author(s): Jérémy Berthelier
Hidetoshi Saze

Last updated by author(s): Apr 18, 2023

Reporting Summary

Nature Portfolio wishes to improve the reproducibility of the work that we publish. This form provides structure for consistency and transparency in reporting. For further information on Nature Portfolio policies, see our Editorial Policies and the Editorial Policy Checklist.

Statistics

For all statistical analyses, confirm that the following items are present in the figure legend, table legend, main text, or Methods section.

n/a	Confirmed	
☐	☒	The exact sample size (*n*) for each experimental group/condition, given as a discrete number and unit of measurement
☐	☒	A statement on whether measurements were taken from distinct samples or whether the same sample was measured repeatedly
☐	☒	The statistical test(s) used AND whether they are one- or two-sided<br>*Only common tests should be described solely by name; describe more complex techniques in the Methods section.*
☒	☐	A description of all covariates tested
☐	☒	A description of any assumptions or corrections, such as tests of normality and adjustment for multiple comparisons
☐	☒	A full description of the statistical parameters including central tendency (e.g. means) or other basic estimates (e.g. regression coefficient) AND variation (e.g. standard deviation) or associated estimates of uncertainty (e.g. confidence intervals)
☐	☒	For null hypothesis testing, the test statistic (e.g. *F*, *t*, *r*) with confidence intervals, effect sizes, degrees of freedom and *P* value noted<br>*Give P values as exact values whenever suitable.*
☒	☐	For Bayesian analysis, information on the choice of priors and Markov chain Monte Carlo settings
☒	☐	For hierarchical and complex designs, identification of the appropriate level for tests and full reporting of outcomes
☐	☒	Estimates of effect sizes (e.g. Cohen's *d*, Pearson's *r*), indicating how they were calculated

Our web collection on statistics for biologists contains articles on many of the points above.

Software and code

Policy information about availability of computer code

Data collection	No software was used for data collection.
Data analysis	ONT basecalling of sequenced DNA and RNA reads were performed using Guppy (v4.4.2)<br>ONT-DRS data were converted from RNA data into cDNA sequences with seqkit (v0.12.1)<br>ONT-DRS data were corrected using LorDEC (v0.9)<br>ONT-DRS data were mapped with minimap2 (v2.17)<br>Transcriptomes were built or merged with Stringtie2 (v2.1.4)<br>was performed with GffCompare<br>A consensus transcriptome was built using Gffcompare (v0.12.6)<br>Detection of ONT-DRS reads with poly(A) tails was done with nanopolish (v0.13.2)<br>DNA methylation from ONT was called with DeepSignal-Plant (v0.1.1)<br>DNA methylation from bisulfite-seq was called with Bismark (v0.23.0)<br><br>Illumina reads were trimmed using fastp (v0.21.0)<br>Genes coverage was analysed with featureCount (v2.0.2)<br>Gene enrichment analysis was conducted online with ShinyGO (v0.75) (http://bioinformatics.sdstate.edu/go/)<br>Illumina reads were mapped with Hisat2 (v2.2.0)<br>Quantification of the expression of transcripts was performed with salmon (v1.3.0)<br>Isoform switch analysis was performed with IsoformSwitchAnalyzeR (v1.6.0) using R (v3.6.1)

Differential expression of isoforms was predicted with DESeq2 (v1.24.0) using R (v3.6.1)

ChIP-seq reads were mapped with Bowtie2 (v2.4.2)
ChIP-seq peak calling was performed with MACS2 (v2.2.7.1)
For visualization of ChIP-seq data, bigWig files were generated with the bamCoverage function of deepTools (v3.4.3)
Overlaps of sets of genomic or transcriptomic features were performed with bedtools (v2.29.2)

The tool ParasiTE was built to detect TE-Gt and can be downloaded on Github (https://github.com/JBerthelier/ParasiTE), and a detailed description of the pipeline can be found in Supplementary Note 1.

For manuscripts utilizing custom algorithms or software that are central to the research but not yet described in published literature, software must be made available to editors and reviewers. We strongly encourage code deposition in a community repository (e.g. GitHub). See the Nature Portfolio guidelines for submitting code & software for further information.

Data

Policy information about availability of data

All manuscripts must include a data availability statement. This statement should provide the following information, where applicable:

- Accession codes, unique identifiers, or web links for publicly available datasets
- A description of any restrictions on data availability
- For clinical datasets or third party data, please ensure that the statement adheres to our policy

The sequencing data generated in this study have been deposited in EMB-EBI European Nucleotide Archive database under acceccion codes PRJEB53848 [https://www.ebi.ac.uk/ena/browser/view/PRJEB53848] (Illumina RNA-seq data, noted that the corresponding Col-0 and ibm2 dataset were previously published26); PRJEB53877 [https://www.ebi.ac.uk/ena/browser/view/PRJEB53877] (ChIP-seq data of epigenetics mutants); PRJEB53877 [https://www.ebi.ac.uk/ena/browser/view/PRJEB53877] (ChIP-seq data of RNA Pol II in ibm2 and edm2); PRJEB53881 [https://www.ebi.ac.uk/ena/browser/view/PRJEB53881] (ONT-DRS data of epigenetics mutants); PRJEB53882 [https://www.ebi.ac.uk/ena/browser/view/PRJEB53882] (ONT DNA of Col-0, met1, and ddm1); PRJEB58752 [https://www.ebi.ac.uk/ena/browser/view/PRJEB58752] (Bisulfite-Seq data of Col-0, ibm1, ibm2 and edm2).

The processed transcriptomes, ONT methylation data, and ChIP-seq data are available at The Plant Epigenetic Database [https://plantepigenetics.oist.jp/].

TAIR10 genome assembly used in this study is available at TAIR website [https://www.arabidopsis.org/download/index-auto.jsp%3Fdir%3D%252Fdownload_files%252FGenes%252FTAIR10_genome_release].
Araport11 transcriptome used in this study is available in Phytozome database [https://data.jgi.doe.gov/refine-download/phytozome?organism=Athaliana&expanded=447].
AtRTD3 transcriptome used in this study is available in at the AtRTD website [https://ics.hutton.ac.uk/atRTD/RTD3].
TAIR10 TE annotation used in this study is available on the URGI Genome Browser [https://urgi.versailles.inra.fr/gb2/gbrowse/tairv10_pub_TEs]

Human research participants

Policy information about studies involving human research participants and Sex and Gender in Research.

Reporting on sex and gender	N.A.
Population characteristics	N.A.
Recruitment	N.A.
Ethics oversight	N.A.

Note that full information on the approval of the study protocol must also be provided in the manuscript.

Field-specific reporting

Please select the one below that is the best fit for your research. If you are not sure, read the appropriate sections before making your selection.

☒ Life sciences ☐ Behavioural & social sciences ☐ Ecological, evolutionary & environmental sciences

For a reference copy of the document with all sections, see nature.com/documents/nr-reporting-summary-flat.pdf

Life sciences study design

All studies must disclose on these points even when the disclosure is negative.

Sample size	No sample size calculation was performed. Sample size was chosen according to the standard in the plant biology field.
Data exclusions	No data exclusion was performed throughout the study.

Replication	Two independent biological replicates for ChiP-seq. Two independent biological replicates for RNA-seq. Three independent biological replicates for ONT-DRS. Four independent biological replicates for qPCR experiments. For pathogen experiment, 70–130 leaves were analyzed per line across three independent experiment replicates. All these replications were successfull.
Randomization	Plant samples were placed randomly in the plant facility.
Blinding	No blinding was applied for sampling. Since most of the data were obtained by bioinformatic analysis with identical parameter settings in our study, blind sampling was not essential.

Reporting for specific materials, systems and methods

We require information from authors about some types of materials, experimental systems and methods used in many studies. Here, indicate whether each material, system or method listed is relevant to your study. If you are not sure if a list item applies to your research, read the appropriate section before selecting a response.

Materials & experimental systems

n/a	Involved in the study
☐	☒ Antibodies
☒	☐ Eukaryotic cell lines
☒	☐ Palaeontology and archaeology
☒	☐ Animals and other organisms
☒	☐ Clinical data
☒	☐ Dual use research of concern

Methods

n/a	Involved in the study
☐	☒ ChIP-seq
☒	☐ Flow cytometry
☒	☐ MRI-based neuroimaging

Antibodies

Antibodies used	Anti-RNA polymerase II CTD repeat YSPTSPS (phospho S2) (Abcam ab5095); Anti-RNA polymerase II CTD repeat YSPTSPS (phospho S5) (Abcam ab5408); Anti-HA antibody (ab9110; abcam).
Validation	Validation of the antibodies were available on the manufacture's web site: Abcam ab5095 (https://www.abcam.co.jp/rna-polymerase-ii-ctd-repeat-ysptsps-phospho-s2-antibody-chip-grade-ab5095.html). Abcam ab5408 (https://www.abcam.co.jp/rna-polymerase-ii-ctd-repeat-ysptsps-phospho-s5-antibody-4h8-chip-grade-ab5408.html). Abcam ab9110 (https://www.abcam.co.jp/ha-tag-antibody-chip-grade-ab9110.html).

ChIP-seq

Data deposition

☒ Confirm that both raw and final processed data have been deposited in a public database such as GEO.

☐ Confirm that you have deposited or provided access to graph files (e.g. BED files) for the called peaks.

Data access links<br>*May remain private before publication.*	All sequencing data have been deposited in EMB-EBI European Nucleotide Archive. The ChIP-seq data of RNA Pol II in Col-0, ibm2, and edm2: PRJEB53877 and PRJEB53880. ChIP-seq data of epigenetic mutants: PRJEB53877.
Files in database submission	chipseq_ibm2_Ser5-RNAPolIII_input_R1.fastq.gz<br>chipseq_ibm2_Ser5-RNAPolIII_input_R2.fastq.gz<br>chipseq_ibm2_Ser5-RNAPolIII_IP1_R1.fastq.gz<br>chipseq_ibm2_Ser5-RNAPolIII_IP1_R2.fastq.gz<br>chipseq_ibm2_Ser5-RNAPolIII_IP2_R1.fastq.gz<br>chipseq_ibm2_Ser5-RNAPolIII_IP2_R2.fastq.gz<br>chipseq_edm2_Ser5-RNAPolII_input_R1.fastq.gz<br>chipseq_edm2_Ser5-RNAPolII_input_R2.fastq.gz<br>chipseq_edm2_Ser5-RNAPolII_IP1_R1.fastq.gz<br>chipseq_edm2_Ser5-RNAPolII_IP1_R2.fastq.gz<br>chipseq_edm2_Ser5-RNAPolII_IP2_R1.fastq.gz<br>chipseq_edm2_Ser5-RNAPolII_IP2_R2.fastq.gz<br>chipseq_ibm2_Ser2-RNAPolIII_input_R1.fastq.gz<br>chipseq_ibm2_Ser2-RNAPolIII_input_R2.fastq.gz<br>chipseq_ibm2_Ser2-RNAPolIII_IP1_R1.fastq.gz<br>chipseq_ibm2_Ser2-RNAPolIII_IP1_R2.fastq.gz<br>chipseq_ibm2_Ser2-RNAPolIII_IP2_R1.fastq.gz<br>chipseq_ibm2_Ser2-RNAPolIII_IP2_R2.fastq.gz<br>chipseq_edm2_Ser2-RNAPolII_input_R1.fastq.gz<br>chipseq_edm2_Ser2-RNAPolII_input_R2.fastq.gz

chipseq_edm2_Ser2-RNAPolII_IP1_R1.fastq.gz
chipseq_edm2_Ser2-RNAPolII_IP1_R2.fastq.gz
chipseq_edm2_Ser2-RNAPolII_IP2_R1.fastq.gz
chipseq_edm2_Ser2-RNAPolII_IP2_R2.fastq.gz
chipseq_IBM2_input1_R1.fastq.gz
chipseq_IBM2_input1_R2.fastq.gz
chipseq_IBM2_input2_R1.fastq.gz
chipseq_IBM2_input2_R2.fastq.gz
chipseq_IBM2_IP1_R1.fastq.gz
chipseq_IBM2_IP1_R2.fastq.gz
chipseq_IBM2_IP2_R1.fastq.gz
chipseq_IBM2_IP2_R2.fastq.gz
chipseq_EDM2_input1_R1.fastq.gz
chipseq_EDM2_input1_R2.fastq.gz
chipseq_EDM2_input2_R1.fastq.gz
chipseq_EDM2_input2_R2.fastq.gz
chipseq_EDM2_IP1_R1.fastq.gz
chipseq_EDM2_IP1_R2.fastq.gz
chipseq_EDM2_IP2_R1.fastq.gz
chipseq_EDM2_IP2_R2.fastq.gz

Genome browser session (e.g. UCSC)

N.A.

Methodology

Replicates

Two replicates for IP in each experiment were performed.

Sequencing depth

Name / Total reads (paired-end)

chipseq_ibm2_Ser5-RNAPolIII_input / 13,474,919
chipseq_ibm2_Ser5-RNAPolII_IP1 / 15,591,630
chipseq_ibm2_Ser5-RNAPolII_IP2 / 16,323,487
chipseq_edm2_Ser5-RNAPolII_input / 11,354,956
chipseq_edm2_Ser5-RNAPolII_IP1 / 13,590,834
chipseq_edm2_Ser5-RNAPolII_IP2 / 14,301,444
chipseq_ibm2_Ser2-RNAPolIII_input / 16,790,781
chipseq_ibm2_Ser2-RNAPolIII_IP1 / 16,205,563
chipseq_ibm2_Ser2-RNAPolIII_IP2 / 18,116,397
chipseq_edm2_Ser2-RNAPolII_input / 17,228,914
chipseq_edm2_Ser2-RNAPolII_IP1 / 15,936,096
chipseq_edm2_Ser2-RNAPolII_IP2 / 19,011,272
chipseq_IBM2_input1 / 19,200,382
chipseq_IBM2_input2 / 16,529,519
chipseq_IBM2_IP1 / 13,544,461
chipseq_IBM2_IP2 / 14,460,862
chipseq_EDM2_input1 / 19,058,766
chipseq_EDM2_input2 / 19,514,511
chipseq_EDM2_IP1 / 15,424,016
chipseq_EDM2_IP2 / 14,207,527

Antibodies

YSPTSPS (phospho S2, ab5095; Abcam, Cambridge, UK)(1: 125 dilution) and anti-RNA polymerase II CTD repeat YSPTSPS (phospho S5, ab5408; Abcam) (1: 125 dilution) antibodies. ChIP-seq for FLAG-HA-IBM2 and EDM2-MYC-HA was performed using an anti-HA antibody (ab9110; abcam) (1: 125 dilution).

Peak calling parameters

ChIP-seq peak calling was performed with MACS2 (v2.2.7.1; parameters: --broad --cutoff-analysis).

Data quality

Data quality was assessed by FastQC, v0.10.1.

Software

ChIP-seq reads were trimmed with fastp (v0.21.0)
ChIP-seq reads were mapped with Bowtie2 (v2.4.2)
ChIP-seq peak calling was performed with MACS2 (v2.2.7.1)
For visualization of ChIP-seq data, bigWig files were generated with the bamCoverage function of deepTools (v3.4.3)
